# Supplementary material for: Development of a core information set for colorectal cancer surgery: a consensus study
Source: BMJ Open. 2019 Nov 14;9(11):e028623. doi: 10.1136/bmjopen-2018-028623 (PMC6886994; doi:10.1136/bmjopen-2018-028623)
Supplement: Supplementary data [file bmjopen-2018-028623supp001.pdf]

Supplementary Table 1 Patient and professional scoring of information domains in Round 1 and 2

	Round 1				Round2			
**Information domain**	**n (%) patients rating domain low importance[a]**	**n (%) patients rating domain high importance[b]**	**n (%) HCP rating domain low importance[a]**	**n (%) HCP rating domain high importance[b]**	**n (%) patients rating domain low importance[a]**	**n (%) patients rating domain high importance[b]**	**n (%) HCP rating domain low importance[a]**	**n (%) HCP rating domain high importance[b]**
Resection margins	2(2)	88(91)	13(13)	69(70)	1(1)	82(94)	5(5)	74(75)
The need for a stoma	3(3)	84(87)	0(0)	97(98)	1(1)	82(94)	0(0)	94(96)
Distant recurrence	8(8)	81(83)	20(20)	48(48)	2(2)	74(85)	7(7)	52(53)
Local recurrence	6(6)	80(82)	21(21)	50(52)	2(2)	73(84)	8(8)	47(48)
Recurrence	7(7)	80(82)	18(18)	52(54)	1(1)	72(83)	8(8)	50(51)
Non-progression	10(10)	80(82)	18(18)	49(50)	2(2)	69(79)	7(7)	50(51)
Disease-free interval	10(10)	79(81)	30(31)	26(27)	4(5)	67(77)	15(15)	21(21)
Sphincter preservation*	14(14)	74(76)						
Lymph node harvest	14(14)	72(74)	56(58)	22(22)	3(3)	55(63)	37(38)	16(16)
Survival	12(12)	71(73)	34(35)	30(31)	6(7)	61(70)	11(11)	30(30)
Physical function	6(6)	70(72)	19(19)	39(39)	2(2)	62(71)	5(5)	25(25)
Problems with the stoma, for example leaks,unpleasant odours/noises or embarrassment	6(6)	66(68)	22(22)	51(53)	2(2)	62(71)	7(7)	37(38)
Overall quality of life	7(7)	64(66)	19(19)	46(46)	4(5)	62(71)	11(11)	42(43)
Role function	6(6)	64(66)	15(15)	49(50)	3(3)	59(68)	7(7)	33(34)
Anastomotic leak	16(16)	63(65)	0(0)	97(98)	5(6)	67(77)	0(0)	94(96)
Ability to cope emotionally with being diagnosed and treated for bowel cancer	9(9)	63(65)	28(29)	28(29)	8(9)	58(67)	18(19)	23(24)
Outlook on life after being diagnosed and treated for bowel cancer	11(11)	63(65)	35(36)	28(29)	7(8)	54(62)	20(20)	28(29)
Self-care	4(4)	62(64)	13(13)	50(52)	0(0)	59(68)	8(8)	36(37)

Rectal bleeding	9(9)	62(64)	32(33)	29(30)	3(3)	57(66)	17(17)	22(23)
General pain	11(11)	61(63)	16(16)	43(43)	7(8)	54(62)	11(11)	32(33)
Deep surgical site infection	18(19)	60(62)	4(5)	81(83)	6(7)	61(70)	0(0)	91(93)
Faecal incontinence	7(7)	59(61)	8(9)	65(66)	0(0)	60(69)	5(5)	67(69)
Overall health	3(3)	59(61)	27(28)	42(42)	5(6)	53(61)	15(15)	25(25)
Problems with stomata (e.g. stenosis, necrosis, retraction)	20(21)	57(59)	9(10)	58(60)	10(11)	57(66)	5(5)	62(64)
Bowel obstruction	9(9)	56(58)	19(19)	41(41)	2(2)	62(71)	6(6)	41(42)
Length of time after surgery until the bowels open	9(9)	56(58)	45(45)	16(16)	4(5)	50(57)	32(33)	12(12)
The need for extra aids to control symptoms, for example pain killers or incontinence pads	14(14)	56(58)	25(26)	35(36)	4(5)	47(54)	11(11)	22(23)
Superficial surgical site infection	17(18)	54(56)	1(2)	77(79)	6(7)	52(60)	1(1)	82(84)
Length of bowel removed	15(15)	54(56)	65(66)	8(9)				
Conversion to open operation	14(14)	53(55)	1(2)	83(85)	8(9)	48(55)	0(0)	76(78)
Diarrhoea	6(6)	52(54)	14(14)	54(56)	5(6)	46(53)	8(8)	40(41)
Faecal urgency	8(8)	51(53)	10(11)	66(67)	3(3)	52(60)	2(2)	69(70)
Constipation	8(8)	51(53)	34(35)	29(30)	6(7)	39(45)	20(20)	12(12)
Ascites	19(20)	51(53)	63(64)	9(10)				
Faecal discrimination	9(9)	50(52)	24(24)	37(37)	4(5)	41(47)	12(12)	37(38)
Length of time after surgery to start eating and drinking	17(18)	50(52)	34(35)	37(37)	7(8)	36(41)	15(15)	21(21)
Cognition	14(14)	50(52)	47(47)	10(11)	10(11)	34(39)	26(26)	11(11)
Wound dehiscence	21(22)	50(52)	24(24)	37(37)				
Enterocutaneous fistula	19(20)	50(52)	40(40)	26(27)				
Fatigue	19(20)	50(52)	41(41)	22(22)				
Length of hospital stay	14(14)	49(51)	26(27)	41(41)	7(8)	39(45)	15(15)	33(34)
Visceral injury	20(21)	47(48)	4(5)	70(71)	4(5)	48(55)	0(0)	76(78)

Sexual function	17(18)	47(48)	2(3)	84(86)	7(8)	48(55)	0(0)	76(78)
ileus	17(18)	47(48)	22(22)	41(41)				
Enterovisceral fistula	20(21)	47(48)	43(43)	23(23)				
Faecal frequency	8(8)	46(47)	8(9)	63(64)	2(2)	40(46)	2(2)	62(64)
Hernia	14(14)	46(47)	23(23)	37(37)				
Tenesmus	16(16)	46(47)	27(28)	38(38)				
Venous thromboembolism	15(15)	45(46)	2(3)	79(81)	4(5)	45(52)	1(1)	72(74)
Delayed wound healing	21(22)	45(46)	28(29)	31(32)				
Emotions – depression	25(26)	45(46)	33(34)	25(26)				
Renal failure	31(32)	44(45)	39(39)	18(18)				
Nausia and vomiting	11(11)	44(45)	35(36)	23(23)				
Urinary incontinence	15(15)	44(45)	40(40)	24(25)				
Operative mortality	34(35)	43(44)	5(6)	81(83)	13(15)	37(43)	2(2)	80(82)
Operative time	18(19)	43(44)	45(45)	14(14)				
Appearance/weight loss	26(27)	43(44)	51(53)	18(18)				
Shortness of breath	18(19)	43(44)	56(58)	10(11)				
Urinary frequency	14(14)	43(44)	49(50)	19(19)				
Reoperation	24(25)	42(43)	13(13)	58(60)	11(13)	33(38)	10(10)	62(64)
Incision size	26(27)	42(43)	35(36)	24(24)				
Dysphagia/regurgitation	19(20)	42(43)	63(64)	5(6)				
Bloating	12(12)	42(43)	58(60)	9(10)				
Dysuria	14(14)	42(43)	62(63)	9(10)				
Emotions – Anxiety	23(24)	42(43)	33(34)	25(26)				
Myocardial infarction	27(28)	40(41)	18(18)	50(52)	12(14)	30(34)	5(5)	45(46)
Finances	32(33)	40(41)	49(51)	22(22)				
Wound seroma	27(28)	39(40)	50(52)	13(13)				
Multi-organ failure	30(31)	39(40)	27(28)	39(39)				
Reflux	21(22)	39(40)	70(71)	5(6)				
Belching, gas or flatulence	18(19)	39(40)	71(72)	4(5)				
Arrhythmia	31(32)	38(39)	46(46)	23(23)				
Non-ischaemic/non-arrhythmic cardiac complications	29(30)	38(39)	45(45)	19(19)				

Cerebrovascular complications	25(26)	38(39)	44(44)	23(23)				
Social function	17(18)	38(39)	51(53)	13(13)				
Septicaemia	30(31)	37(38)	29(30)	30(31)				
How long it takes for a bowel movement	19(20)	37(38)	59(61)	8(9)				
postoperative haemorrhage	31(32)	36(37)	7(8)	75(77)	17(20)	32(37)	1(1)	75(76)
Pneumonia	27(28)	36(37)	9(10)	53(55)	12(14)	27(31)	3(3)	41(42)
Urinary tract infection	30(31)	35(36)	43(43)	21(21)				
Feeling faint	24(25)	35(36)	71(72)	7(8)				
Anorexia	24(25)	35(36)	52(54)	15(15)				
Insomnia	20(21)	34(35)	68(69)	9(10)				
Non-infective respiratory complications	32(33)	33(34)	39(39)	19(19)				
Thrombophlebitis	35(36)	31(32)	54(56)	13(13)				
Operative blood loss	33(34)	30(31)	31(32)	41(41)	20(23)	22(25)	13(14)	30(30)
Unplanned readmission	31(32)	30(31)	33(34)	29(30)	13(15)	16(18)	16(16)	23(24)
Allergic reactions	29(30)	29(30)	67(68)	13(13)				
Catheter complications	35(36)	29(30)	54(56)	15(15)				
Problems with periods (female)	9(9)	27(28)	59(61)	15(15)				
Rash or other skin problems	38(39)	22(23)	84(86)	1(2)				
Equipment failure	43(44)	21(22)	64(65)	11(12)				
Spiritual or faith issues	46(47)	13(13)	68(69)	8(9)				
The need for adjuvant chemotherapy#			4(5)	82(84)	1(1)	77(89)	2(2)	79(80)
Stoma reversal#			0(1)	79(81)	1(1)	73(84)	1(1)	82(84)
Expected in hosptial experience#			4(5)	80(82)	7(8)	70(80)	0(0)	80(82)
The need for bowel preparation#			11(12)	64(65)	5(6)	53(61)	7(7)	65(66)
Family risk of bowel cancer#			19(19)	42(42)				

[a]Low importance is defined as scoring 1-3 on a nine-point Likert scale

[b]High importance is defined as scoring 7-9 on a nine-point Likert scale

*Patient feedback resulted in the combination of domains “Sphincter preservation” into “Stoma rates” in Round 1, as these concepts were considered synonymous. “Sphincter preservation” was therefore not included in the Round 1 professional questionnaire.

#Patient feedback resulted in the addition of five information domains included in the Round 1 professional questionnaire
