# Supplementary material for: Development of a core information set for colorectal cancer surgery: a consensus study
Source: BMJ Open. 2019 Nov 14;9(11):e028623. doi: 10.1136/bmjopen-2018-028623 (PMC6886994; doi:10.1136/bmjopen-2018-028623)
Supplement: Author's manuscript [file bmjopen-2018-028623.draft_revisions.pdf]

BMJ Open is committed to open peer review. As part of this commitment we make the peer review history of every article we publish publicly available.

When an article is published we post the peer reviewers' comments and the authors' responses online. We also post the versions of the paper that were used during peer review. These are the versions that the peer review comments apply to.

The versions of the paper that follow are the versions that were submitted during the peer review process. They are not the versions of record or the final published versions. They should not be cited or distributed as the published version of this manuscript.

BMJ Open is an open access journal and the full, final, typeset and author-corrected version of record of the manuscript is available on our site with no access controls, subscription charges or pay-per-view fees (http://bmjopen.bmj.com).

If you have any questions on BMJ Open's open peer review process please email info.bmjopen@bmj.com

BMJ Open

The development of a core information for colorectal cancer surgery: a consensus study

Journal:	*BMJ Open*
Manuscript ID	bmjopen-2018-028623
Article Type:	Research
Date Submitted by the Author:	17-Dec-2018
Complete List of Authors:	McNair, Angus; University of Bristol, Centre for Surgical Research, Bristol Medical School: Population Health Sciences; North Bristol NHS Trust, GI Surgery<br>Whistance, Robert; University of Bristol, Centre for Surgical Research, Bristol Medical School: Population Health Sciences<br>Main, Barry; University of Bristol, Centre for Surgical Research, Bristol Medical School: Population Health Sciences<br>Forsythe, Rachael<br>Macefield, Rhiannon; University of Bristol, Centre for Surgical Research, Bristol Medical School: Population Health Sciences<br>Rees, Jonathan; University Hospitals Bristol NHS Foundation Trust, Division of Surgery,; University of Bristol, Centre for Surgical Research<br>Pullyblank, Anne; West of England Academic Health Science Network; North Bristol NHS Trust<br>Avery, Kerry; University of Bristol, Centre for Surgical Research, Bristol Medical School: Population Health Sciences<br>Brookes, Sara; Institute of Cancer and Genomic Sciences, University of Birmingham, Cancer Research UK Clinical Trials Unit ; University of Bristol, Population Health Sciences<br>Thomas, Michael; University Hospitals Bristol NHS Foundation Trust, Division of Surgery,<br>Sylvester, Paul; University Hospitals Bristol NHS Foundation Trust, Division of Surgery,<br>Russell, Ann; National Cancer Research Institute, Consumer Liaison Group<br>Oliver, Alfred; National Cancer Research Institute, Consumer Liaison Group<br>Morton, Dion; University of Birmingham<br>Kennedy, Robin; St Mark's Hospital, Department of Surgery<br>Jayne, David; Leeds Teaching Hospitals,<br>Huxtable, Richard; University of Bristol, Bristol Medical School: Population Health Sciences<br>Hackett, Roland; South West Cancer Network, Colorectal Site Specific Group<br>Dutton, Susan; University of Oxford, CSM<br>Coleman, Mark; University Hospitals Plymouth NHS Trust, Derriford Hospital, Department of Surgery<br>Card, Mia; University Hospitals Bristol NHS Foundation Trust, Division of Surgery,<br>Brown, Julia; University of Leeds, Consultant Leeds Institute of Clinical Trials Research

	Blazeby, Jane; University of Bristol, Centre for Surgical Research, Bristol Medical School: Population Health Sciences

Keywords:	Colorectal surgery < SURGERY, MEDICAL ETHICS, Gastrointestinal tumours < ONCOLOGY

SCHOLARONE™ Manuscripts

The development of a core information for colorectal cancer surgery: a consensus study

Angus GK McNair PhD FRCS[1,3], Robert N Whistance MRCS[1,2], Barry Main PhD[1,4], Rachael O Forsythe MRCS[1,2], Rhiannon Macefield[1], Jonathan Rees PhD[1], Anne M Pullyblank FRCS[3], Kerry NL Avery PhD[1], Sara T Brookes PhD[1], Michael G Thomas FRCS[5], Paul A Sylvester FRCS[5], Ann Russell[6], Alfred Oliver[6], Dion Morton FRCS[7], Robin Kennedy FRCS[8], David G Jayne FRCS[9], Richard Huxtable PhD[10], Roland Hackett[11], Susan J Dutton PhD[12], Mark G Coleman FRCS[13], Mia Card[5], Julia Brown PhD[14], Jane M Blazeby FRCS[1,2]

[1]Centre for Surgical Research, Bristol Medical School: Population Health Sciences, University of Bristol, 39 Whatley Road, Bristol, UK

[2]Division of Surgery Head and Neck, University Hospitals Bristol NHS Foundation Trust, Bristol, UK

[3]Department of General Surgery, North Bristol NHS Trust, Bristol, UK

[4]Severn School of Surgery, Bristol, UK

[5]Colorectal Surgery Unit, University Hospitals Bristol NHS Foundation Trust, Bristol, UK

[6]Colorectal Consumer Liaison Group, National Cancer Research Institute, London, UK

[7]Academic Department of Surgery, University of Birmingham, Birmingham, UK

[8]Department of Surgery, St Mark's Hospital and Academic Institute, Harrow, UK

[9]Academic Surgical Unit, St James' University Hospital NHS Trust, Leeds, UK

[10]Centre for Ethics in Medicine, University of Bristol, Bristol, UK

[11] Colorectal Site Specific Group, Somerset, Wiltshire, Avon & Gloucestershire, South West Cancer Network UK

[12]Centre for Statistics in Medicine and Oxford Clinical Trials Research Unit, Nuffield Department of Orthopaedics, Rheumatology and Musculoskeletal Sciences, University of Oxford, Oxford, UK

[13]Department of Colorectal Surgery, Plymouth Hospitals NHS Trust, Plymouth, UK

[14]Clinical Trials Research Unit, University of Leeds, Leeds, UK

Corresponding author:
Angus McNair
Centre for Surgical Research
Bristol Medical School: Population Health Sciences
University of Bristol
Canynge Hall
39 Whatley Road
Bristol
BS8 2PS United Kingdom
Email: angus.mcnair@bristol.ac.uk
Tel: +44 177 928 3495 Fax: +44 117 925 2736

Funding:
This work was supported by the MRC ConDuCT-II Hub (Collaboration and innovation for Difficult and Complex randomised controlled Trials In Invasive procedures - MR/K025643/1) and the NIHR Biomedical Research Centre at the University Hospitals Bristol NHS Foundation Trust and the University of Bristol. RNW was supported by an NIHR doctoral research fellowship. AM is funded by a Clinician Scientist Fellowship (NIHR-CS-2017-17-010) from the National Institute for Health Research. The views expressed in this publication are those of the author(s) and not necessarily those of the NHS, the National Institute for Health Research, Health Education England or the Department of Health

For peer review only - http://bmjopen.bmj.com/site/about/guidelines.xhtml

ABSTRACT

Objective: 'Core information sets' (CIS) represent baseline information, agreed by patients and professionals, to stimulate individualized patient-centred discussions. This study developed a CIS for use before colorectal cancer (CRC) surgery.

Design: Three phase consensus study: 1) Systematic literature reviews and patient interviews to identify potential information of importance to patients; 2) UK national Delphi survey of patients and professionals to rate the importance of the information; 3) International consensus meeting to agree the final CIS.

Setting: National United Kingdom CRC centres.

Participants: Purposive sampling was conducted to ensure CRC center representation based upon geographical region and caseload volume. Responses were received from 63/81(78%) centers (90 professionals). Adult patients who had undergone CRC surgery were eligible, and purposive sampling was conducted to ensure representation based on age, sex and cancer location (rectum, left and right colon). Responses were received from 97/267(35%) patients with a wide age range(29-87), equal sex ratio and cancer location. Attendees of the international Tripartite Colorectal Conference were eligible for the consensus meeting

Outcomes: Phase 1: Information of potential importance to patients was extracted verbatim and operationalized into a Delphi questionnaire. Phase 2: Patients and professionals rated the importance information on a 9-point Likert scale, and re-surveyed following group feedback. Information rated of low importance were discarded using pre-defined criteria. Phase 3: A modified nominal group technique was used to gain final consensus in separate consensus meetings with patients and professionals.

Results: Data sources identified 1216 pieces of information that informed a 100-item questionnaire. Analysis led to 46 and 23 information domains being retained after the first and second surveys, respectively. The final CIS included 11 concepts including specific surgical complications, short and long-term survival, disease recurrence, stoma and quality of life issues.

For peer review only - http://bmjopen.bmj.com/site/about/guidelines.xhtml

Conclusions: This study has established a CIS for professionals to discuss with patients before CRC surgery.

Strengths as limitations of this study

- Robust consensus methodology and transparent reporting was used to allow public scrutiny of all information included or excluded from the CIS
- Patient and professional views were given equal weighting in the consensus process
- The patient response rate was low, however, purposive sampling ensured representation of key demographics.

Keywords: Colorectal cancer, Communication, informed consent, shared decision-making

Word Count: 3236

For peer review only - http://bmjopen.bmj.com/site/about/guidelines.xhtml

Introduction

High-quality, patient-centered communication is a cornerstone of clinical practice in the United Kingdom and worldwide[1-4]. Such communication can facilitate shared decision making, where doctors and patients agree on a treatment plan that best fits the patient's needs, and ensure that patients' fundamental ethical right to self-determination is maintained through the process of informed consent[5]. This is particularly important in surgical oncology. For many patients with cancer, surgery offers the best chance of long-term survival but this must be balanced against short term mortality and potentially irreversible deterioration in quality of life. Understanding and weighing-up the nature and consequences of treatment, based upon personal values, is therefore an important part of the cancer journey.

There are, however, significant challenges in addressing the information needs of individual patients. The amount of information that could be discussed is vast, and it is unclear what information is critical to inform understanding in an individual. Generally patients prefer more rather than less information[6], but there is a danger of overwhelming patients with information that is not important to them, and this may reduce understanding and increase anxiety[7]. Patient-led communication, where discussions are guided by the individual, is helpful but patients may lack sufficient baseline knowledge to ask important questions.

Beyond these professional and ethical responsibilities, there are additional requirements to meet legal standards of information provision for informed consent. There has been a gradual shift away from a physician-centered, paternalistic model of consent towards a "reasonable-patient" standard[5]. This aligns the process of informed consent to the patients' perspective, and requires surgeons to discuss all relevant information about a proposed treatment that an objective patient would find material to making an informed decision. This model has been endorsed through the recent *Montgomery* ruling in the UK, and is common in other jurisdictions[8], but it is at once confusing and

For peer review only

helpful in addressing patients' information needs[9]. On the one hand, it is a somewhat abstract concept that does little to help physicians tailor information to the individual[10]. Without a clear understanding of who a reasonable patient might be, the temptation exists to over disclose. On the other hand, the reasonable-patient standard might serve best if viewed as a baseline from which more meaningful, person-centered conversations develop.

One method for balancing over and under disclosure of information is to develop a "core information set" for a specific treatment. Core information represents baseline information, determined by patients and clinicians, necessary to stimulate further patient-centered communication[11-14]. A core information set is intended for use once a treatment recommendation or decision has been made and will provide relevant information about a single intervention. This differs from the role and purpose of a decision aid, which deliberately provides information about alternative treatment choices[15]. It has the advantages of being feasible and transferable to a wide number of settings, possible to define using established health services research methodology, aligned with contemporary ethical theory, and potentially meeting the "reasonable patient" legal standard of IC. CISs are available for oesophageal and oropharyngeal cancers [12 16].

The aim of this study is to define a core information set for CRC surgery. CRC is the third most common cancer in men and second most common in women worldwide, with an estimated incidence over 740,000 and 610,000 respectively[17]. The majority of these patients will undergo a surgical resection and methods are therefore needed to ensure that informed consent is optimized.

Methods

Study Design

This study was conducted in three phases using methods modified from the development of core outcome sets for randomized controlled trials[18]: 1) A long-list of potential information of importance before CRC surgery was identified, and categorized into domains; 2) Domains were operationalized

For peer review only - http://bmjopen.bmj.com/site/about/guidelines.xhtml

into a questionnaire that was used to survey stakeholders' views on the importance of each domain using Delphi methods; 3) Consensus meetings with patients and professionals were used to finalize the CIS. Ethics regulatory approval was granted (UK NRES 10/H0102/82).

This study was conducted in parallel with the development of a set of outcomes to measure in CRC randomized trials, the results of which are published elsewhere[19]. The same study population was used to concurrently address this separate research question. It was hypothesized that there may be observed differences between the core outcome and information sets as they are conceptually different.

Phase 1 - Domain generation

Information about CRC surgery was identified from systematic reviews of clinical and patient reported outcome literature (published elsewhere[20 21]), supplemented by a review of patient information leaflets and interviews with patients to identify additional information not present in the published literature.

An information long-list was created, and similar information was categorized into domains by two members of the study team and a patient representative. The final domains were operationalized into questionnaire items using lay language, and piloted by patients for face validity, understanding and acceptability.

Phase 2 - Delphi consensus process

The questionnaire was sent to key stakeholders (CRC surgeons, specialist nurses and patients who had undergone surgery for CRC). Patients were essential stakeholders as they are the recipient of treatment, and surgeons and nurses have an in-depth understanding of the potential impact of surgery.

For peer review only - http://bmjopen.bmj.com/site/about/guidelines.xhtml

Professionals were identified from UK National Health Service hospital trusts that participated in the UK National Bowel Cancer Audit. Non-probabilistic purposive sampling was conducted to ensure center variation based upon geographical region (Northern England, the Midlands, South West and South East England, and Wales), and caseload volume. Patients were recruited from University Hospitals Bristol NHS Foundation Trust, North Bristol NHS Trust and Plymouth Hospitals NHS trust. Participants were approached by post and sent the questionnaire with a stamp addressed return envelope. Non-probabilistic purposive sampling was conducted to ensure representation based on age, sex and cancer site (rectum, left colon, right colon). Demographic data were collected including area of deprivation, marital status, employment status, educational level. Deprivation was as defined by the UK Office of National Statistics Index of Multiple Deprivation at lower layer Super Output Area level for the individual[22]. This is a combined measure of income, employment, health and disability, education, barriers to public services, crime and living environment. Educational level was defined as up to basic education (to the age of 16 or completion of the UK General Certificate of Secondary Education or equivalent), further education (subsequent qualifications to the age of 18), undergraduate and postgraduate education.

Questionnaires asked participants to rate the importance of information domains on a nine-point Likert scale, ranging from 1 (‘not essential’) to 9 (‘absolutely essential’). Information domains considered not essential after round 1 were discarded (see analyses, below). In round 2, participants were provided with feedback from round 1 in the form of their previous score for each domain and a mean score from their stakeholder group. Participants then rescored each information domain on the nine-point Likert scale, and the results used to determine which domains should be retained and presented in the consensus meetings.

Professionals were also asked to rate the importance of most domains to be included as trial outcome measures in a core outcome set, the results of which are published elsewhere[19]. An example page from the questionnaire is presented in Figure 1 to demonstrate how professionals

For peer review only - http://bmjopen.bmj.com/site/about/guidelines.xhtml

were asked the both questions simultaneously. Some information did not have a corresponding trial outcome measure (e.g. "expected in hospital experience" or "family risk of bowel cancer") and, in those instances, professionals were only asked to rate them as information. Patients were not asked separate questions about trial outcome measures because, based on feedback from the patient steering group, the two concepts were considered synonymous.

Phase 3 – Face to face consensus meetings

Separate consensus meetings were held with professionals and patients. The professional consensus meeting was conducted at the Tripartite Colorectal Meeting (meeting of the Association of Coloproctology of Great Britain and Ireland, the American Society of Colon and Rectal Surgeons, the Royal Society of Medicine, Royal Australasian College of Surgeons, Colorectal Surgical Society of Australia and New Zealand and the European Society of Coloproctology) in Birmingham, UK in 2014. The meeting was open to all members of international societies. The patient meeting was held in Bristol, UK in 2013. Attendees at this meeting were all from the UK and had completed the questionnaire surveys and responded to an invitation to attend a consensus meeting.

The retained information domains from the second survey were presented and discussed at the meetings. Anonymized voting took place to ask participants to vote each domain as either 'In', 'Out' or 'Unsure' using electronic keypads. Histograms and descriptive statistics were created for each domain during the meeting and displayed to the participants. Where consensus was not reached further discussion ensured and additional voting.

Sample size

There are no agreed methods to set the sample size for Delphi surveys or consensus meetings. Therefore, an opportunistic approach was used with the aim of obtaining approximately 100 respondents for each stakeholder group for the survey and a smaller group in which discussion could take place in the consensus meetings.

For peer review only - http://bmjopen.bmj.com/site/about/guidelines.xhtml

Data analyses

Information domains rated between 7-9 by over 50%, and between 1-3 by less than 15%, of respondents in round 1 were retained for round 2. Information domains not meeting these criteria were discarded. Mean scores were calculated for each retained domain to form the feedback for Round 2. Round 2 domains were retained using stricter criteria (between 7-9 by over 70%, and between 1-3 by less than 15%, of respondents). There are no agreed methods for selecting cut-off criteria within Delphi studies[23], and therefore the criteria were selected after discussion with collaborators. Domains retained after Round 2 were considered in the consensus meetings. Inclusion in the final CIS was determined at the professional consensus meeting by majority vote. In the patient consensus meeting, two rounds of voting were conducted to ensure patients adequately understood the domains. Domains that were voted for by 60% of patients were included in the CIS after initial voting. Those voted for by 40-60% were brought forward for further discussion and those voted for my less than 40% were discarded. In the second round, domains that achieved a two-thirds majority were included. All information domains retained from either meetings were included in the final CIS. Patients considered important information for informed consent and trial outcomes to be synonymous. Results of the patient consensus meeting are therefore presented as part of the core outcome set and are not repeated here[19].

Patient and public involvement

Patients collaborators were involved in developing the study design, categorizing information from data sources into domains, designing the Delphi questionnaire using patient centred language, and interpreting the clinical significance of results.

Data statement

Data from Phase 1 including full domain categorization is available open access[20 21]. Results of the development of the core outcome set are similarly available[19].

For peer review only - http://bmjopen.bmj.com/site/about/guidelines.xhtml

Results

Phase 1 - Domain generation

All data sources identified 1216 pieces of information on CRC surgery that were categorized into 100 domains. A summary of the results is presented in Figure 2.

Phase 2 - Delphi consensus process

A total of 63/81 (78%) CRC centers responded including 90 surgeons and 8 specialist nurses (Table 1). Centers represented all geographical regions of England and Wales, and caseload ranged from 38-275 operations per annum. Patient response rate was 97/267 (36%). The age range was wide, sex ratio equal and similar numbers of patients had rectal, left and right colonic tumors. Many patients lived in areas of low deprivation but there was an even distribution of basic and higher educational level. Professionals rated information about short-term technical outcomes of greatest importance in Round 1 including anastomotic leak, adequacy of resection margins and peri-operative mortality (supplementary Table 1). Although these issues were also important to patients, a priority was given to information about longer-term outcomes such as survival, distant recurrence and impact on quality of life. A total of 46 domains were retained for Round 2.

The response rate in Round 2 was 75% (78/104) for health professionals and 90% (87/97) for patients. The provision of feedback and more stringent cut-off criteria in Round 2 resulted in 23 domains being retained for consideration in the consensus meetings.

Phase 3 - consensus meetings

The professional and patient consensus meetings were attended by 61 and 14 participants respectively. Anonymized voting reached a consensus on six domains at the professionals meeting (Table 2). In initial anonymized voting at the patient consensus meeting, 11 domains were voted 'in', three were voted 'out' and nine were inconclusive. Subsequently, domains that were recognized as

For peer review only - http://bmjopen.bmj.com/site/about/guidelines.xhtml

overlapping were combined[19]. ‘Length of hospital stay’ was broadened in scope to include all details of patients’ pathway through hospital such as the location of the hospital and ward, anticipated length of hospital stay and follow-up arrangements. This information domain was re-named ‘Expected in-hospital experiences’. A second round of voting reached a consensus on including three more domains into the CIS: ‘anastomotic leak’, ‘stoma complications’ and ‘sexual functioning’. Patient and professional CIS were then combined (Table 3), creating a final list of 11 domains.

Discussion:

This study developed a CIS to use to inform consent for CRC surgery. A wide range of sources including published studies and patient interviews were used to identify the initial long-list of 100 information domains that could be communicated to patients. Established consensus methods were used to prioritize the views of patients and professionals to identify 23 domains of the greatest importance. Finally, consensus meetings with an international group of surgeons and UK patients agreed on the final CIS. It is recommended that the domains in the information set are discussed with all patients before CRC surgery as a baseline to improve patient understanding of expected events and outcomes of surgery and to catalyze questions relevant to the patient for further discussion.

Cancer patients’ information needs have been studied extensively[6 24]. Whilst these papers raise important issues, none have examined in detail the information both patients and professionals consider essential to communicate in advance of surgery for CRC. A systematic review identified 239 studies investigating the information needs of patients[24]. It used a qualitative framework analysis to chart patients’ documented information needs across studies. “Treatment-related information” was the most commonly documented need (561 mentions (26%) out of a total 2122 in 239 studies), of which information about “risks and benefits of treatment” was the most frequent. “Rehabilitation information” (384 (18%) mentions), specifically “stoma care” was the next most frequently

For peer review only - http://bmjopen.bmj.com/site/about/guidelines.xhtml

documented need. A Dutch cross sectional survey asked 101 surgeons (response rate 43%) to rate the importance of 12 selected items of information about risks of anastomotic leak and stomata[25]. Most surgeons reported to "always" provide information about the risk of anastomotic leak (99%), re-operation (93%) and stoma (93%). It is not clear, however, how this questionnaire was developed, and the scope was limited when compared to the 100 information domains included in this study. Nonetheless, these results triangulate well with the findings of the core information set work presented here.

Although there are no published core information sets in CRC surgery, a similar concept has been developed for patients undergoing radiotherapy for rectal cancer[26]. A total of 37 information domains were identified through analysis of audio recorded clinical consultations and systematic literature reviews. Delphi surveys and consensus meetings with groups of patients and radiation oncologists gained consensus on 13 benefits and harms of treatment that should be discussed in consultations. Some of this information was found to have similar importance with surgeons and patients undergoing CRC surgery including information about local cancer control, survival, sexual and bowel function. Understandably, other information is unique to surgical therapy. Indeed a CIS has been developed for patients undergoing oesophagectomy[12], which shares several characteristics with the colorectal CIS, such as recommending discussions about in-hospital mortality and major complications. Differences in quality of life information reflect the nature of the surgeries, with the colorectal CIS including issues of defecation, whereas the esophageal CIS recommends discussing eating. In addition, patients and professionals agreed that discussing stomata was important before colorectal but not esophageal surgery where esophagostomy is infrequently required. It is possible that future CIS in other areas of surgical oncology will be developed and generic issues identified across the sets relevant to all patients with cancer.

Strengths and Limitations

For peer review only - http://bmjopen.bmj.com/site/about/guidelines.xhtml

Robust consensus methodology and established guidelines modified from the development of core outcome sets for randomized trials were used to develop this CIS, but there are some weaknesses. The large amount of information identified in Phase 1 required the grouping of information into domains. This introduces an element of subjectivity that was minimized through independent dual categorization, however, information may have been inappropriately grouped or separated. For example, the Dutch CIS for rectal cancer radiotherapy recommended the discussion of five topics around sexual function that this study had combined into one domain[26]. Of note, patients in this study had the opportunity to separate concepts out of domains in the consensus meetings but none chose to do so. Conversely, several domains were amalgamated where participants considered that they were unnecessarily detailed. In addition, patients were involved with the information categorization process and agreed on the domains in advance of the Delphi process. In phase 2, the scope of the Delphi process was limited to the UK before the CIS development process was opened internationally to professionals in Phase 3. This was done to exclude the least important information without the complexity of a multinational Delphi process, however different domains may have been brought forward for discussion at the consensus meetings if this were conducted. It will therefore be important to validate this set in other cultures.

There are no definitive guidelines on sample size and response rates for Delphi studies. The total number of participants in this study is comparatively high and there was a good representation of UK CRC surgical centers, but the response rates from patients was much lower. The effect of this on the validity of the Delphi is unclear because the methodology does not require a representative sample, but to gain a consensus among a wide range of individuals with disparate opinions. In that respect, this study achieved wide diversity based on a priori patient characteristics.

This study has identified an agreed minimum standard of information to be communicated before CRC surgery. Further research is now required to investigate methods to communicate this information effectively in routine practice. This may include agenda setting in clinical encounters[27],

For peer review only - http://bmjopen.bmj.com/site/about/guidelines.xhtml

using visual communication aids[28 29], or modifying hospital information leaflets to detail core information. Recent reviews of interventions to improve informed consent, however, showed that most were inadequately developed, without theory or conceptualisation[30 31]. Included studies were poorly designed, susceptible to bias, and outcome measures to assess interventions for IC were inconsistent. It will be necessary to pilot core information carefully, adhering to guidelines for the development of complex interventions[32], to ensure it is ready for robust evaluation in a cluster randomized trial. Further research is also needed to understand how CISs relate back to modern professional, ethical and legal standards worldwide.

In conclusion, this study developed an evidence-based core information set to communicate to patients before CRC surgery. It is not intended to replace individualized discussions with patients, but to act as a consistent starting point to catalyze further patient-centered discussions. Core information can be communicated by any professional in any healthcare setting, and form the basis of high-quality written information leaflets. This coordinated and reliable approach to information provision may help patients gain sufficient understanding to undergo surgery for CRC in a post *Montgomery* era.

Acknowledgements

We would like to thank Neil Smart and Katherine Baker for their help running the consensus meetings, and Claudette Blake for her administrative support throughout the whole project.

Author Contributions

Wrote the first draft of the manuscript: AGKM RNW RM JR JMB. Contributed to the writing of the manuscript: AGKM RNWROF RM JR AMP MGT AR DM SJD JB KNLA STB JMB.

Agree with the manuscript's results and conclusions: AGKM RNW ROF RM JR AMP MGT PAS AR AO DM RK DGJ RHu RHa SJD MGC MC JB KNLA STB JMB. All authors have read, and confirm that they meet, ICMJE criteria for authorship.

For peer review only - http://bmjopen.bmj.com/site/about/guidelines.xhtml

Disclosure: AM is funded by a Clinician Scientist Fellowship (NIHR-CS-2017-17-010) from the UK National Institute for Health Research (NIHR) and supported by the NIHR Biomedical Research Centre at the University Hospitals Bristol NHS Foundation Trust and the University of Bristol. The views expressed in this publication are those of the author(s) and not necessarily those of the NHS, the National Institute for Health Research, Health Education England or the Department of Health

Conflict of Interest

None declared

For peer review only

For peer review only - http://bmjopen.bmj.com/site/about/guidelines.xhtml

References

1. Epstein RM, Street RL. Patient-Centered Communication in Cancer Care: Promoting healing and reducing suffering. Bethesda, MD: National Cancer Institute, 2007.
2. Department of Health. The NHS constitution for England: Department of Health, 2009.
3. Australian Commission on Safety and Quality in Health Care. Patient centred care: Improving quality and safety through partnerships with patients and consumers. 2011. http://www.safetyandquality.gov.au/wp-content/uploads/2012/03/PCC_Paper_August.pdf (accessed 03/10/2016).
4. United States Government Publishing Office. The Patient Protection and Affordable Care Act. Washington DC: United States Government Office, 2010.
5. Spatz ES, Krumholz HM, Moulton BW. The New Era of Informed Consent: Getting to a Reasonable-Patient Standard Through Shared Decision Making. *JAMA* 2016;315(19):2063-4. doi: 10.1001/jama.2016.3070
6. Rutten LJ, Arora NK, Bakos AD, et al. Information needs and sources of information among cancer patients: a systematic review of research (1980-2003). *Patient Educ Couns* 2005;57(3):250-61. doi: 10.1016/j.pec.2004.06.006
7. Leydon GM, Boulton M, Moynihan C, et al. Cancer patients' information needs and information seeking behaviour: in depth interview study. *BMJ* 2000;320:909-13. doi: 10.1136/bmj.320.7239.909
8. King JS, Moulton BW. Rethinking informed consent: The case for shared medical decision-making. *Am J Law Med* 2006;32(4):429-501.
9. Main BG, McNair A, Blazeby JM. Informed Consent and the Reasonable-Patient Standard. *JAMA* 2016;316(9):992-3. doi: 10.1001/jama.2016.10555
10. Montgomery J, Montgomery E. Montgomery on informed consent: an inexpert decision? *J Med Ethics* 2016;42(2):89-94. doi: 10.1136/medethics-2015-102862 [published Online First: 2016/01/27]
11. Beauchamp TL, Childress JF. Respect for autonomy. Principles of Biomedical Ethics. Oxford: Oxford University Press 2001:57-112.
12. Blazeby JM, Macefield R, Blencowe NS, et al. Core information set for oesophageal cancer surgery. *Br J Surg* 2015;102(8):936-43. doi: 10.1002/bjs.9840
13. Main BG, Davies L, McNair A, et al. Bringing Informed Consent Back to Patients. *British Medical Journal* 2015. http://blogs.bmj.com/bmj/2014/08/05/barry-main-et-al-bringing-informed-consent-back-to-patients (accessed 07/04/2015).
14. Main BG, McNair AGK, Huxtable R, et al. Core information sets for informed consent to surgical interventions: baseline information of importance to patients and clinicians. *BMC Med Ethics* 2017;18(1):29. doi: 10.1186/s12910-017-0188-7 [published Online First: 2017/04/28]
15. Agoritsas T, Heen AF, Brandt L, et al. Decision aids that really promote shared decision making: the pace quickens. *BMJ* 2015;350:g7624. doi: 10.1136/bmj.g7624
16. Main BG, McNair AGK, Haworth S, et al. Core information set for informed consent to surgery for oral or oropharyngeal cancer: A mixed-methods study. *Clin Otolaryngol* 2018;43(2):624-31. doi: 10.1111/coa.13037 [published Online First: 2017/11/28]
17. Cancer IAfRo. GLOBOCAN 2012: Estimated Cancer Incidence, Mortality and Prevalence Worldwide in 2012. 2012. http://globocan.iarc.fr/Pages/fact_sheets_cancer.aspx (accessed 17/07/2015).

For peer review only - http://bmjopen.bmj.com/site/about/guidelines.xhtml

18. Williamson PR, Altman DG, Blazeby JM, et al. Developing core outcome sets for clinical trials: issues to consider. *Trials* 2012;13:132. doi: 10.1186/1745-6215-13-132
19. McNair A, Whistance RN, Forsythe RO, et al. Core Outcomes for Colorectal Cancer Surgery: A Consensus Study. *PLoS Med* 2016;In press
20. McNair AG, Whistance RN, Forsythe RO, et al. Synthesis and summary of patient-reported outcomes (PRO) to inform the development of a core outcome set in colorectal cancer surgery. *Colorectal Dis* 2015:In press.
21. Whistance RN, Forsythe RO, McNair AG, et al. A systematic review of outcome reporting in colorectal cancer surgery. *Colorectal Dis* 2013;15(10):e548-60. doi: 10.1111/codi.12378
22. McLennan D, Barnes H, Noble M, et al. The English Indices of Deprivation 2010. 2011. https://www.gov.uk/government/uploads/system/uploads/attachment_data/file/6320/1870718.pdf (accessed 27/07/2015).
23. Black N, Murphy M, Lamping D, et al. Consensus development methods: a review of best practice in creating clinical guidelines. *Journal of health services research & policy* 1999;4(4):236-48.
24. van Mossel C, Leitz L, Scott S, et al. Information needs across the colorectal cancer care continuum: scoping the literature. *Eur J Cancer Care (Engl)* 2012;21(3):296-320. doi: 10.1111/j.1365-2354.2012.01340.x
25. Snijders HS, Kunneman M, Bonsing BA, et al. Preoperative risk information and patient involvement in surgical treatment for rectal and sigmoid cancer. *Colorectal Dis* 2014;16(2):O43-9. doi: 10.1111/codi.12481
26. Kunneman M, Pieterse AH, Stiggelbout AM, et al. Which benefits and harms of preoperative radiotherapy should be addressed? A Delphi consensus study among rectal cancer patients and radiation oncologists. *Radiother Oncol* 2015;114(2):212-7. doi: 10.1016/j.radonc.2014.11.034
27. Gobat N, Kinnersley P, Gregory JW, et al. What is agenda setting in the clinical encounter? Consensus from literature review and expert consultation. *Patient Educ Couns* 2015;98(7):822-9. doi: 10.1016/j.pec.2015.03.024
28. McNair AG, Brookes ST, Davis CR, et al. Communicating the results of randomized clinical trials: do patients understand multidimensional patient-reported outcomes? *J Clin Oncol* 2010;28(5):738-43.
29. Davis CR, McNair AG, Brigic A, et al. Optimising methods for communicating survival data to patients undergoing cancer surgery. *Eur J Cancer* 2010;46(18):3192-99.
30. Kinnersley P, Phillips K, Savage K, et al. Interventions to promote informed consent for patients undergoing surgical and other invasive healthcare procedures. *Cochrane Database of Systematic Reviews* 2013(7) doi: Artn Cd009445
Doi 10.1002/14651858.Cd009445.Pub2
31. Kim S, Jabori S, O'Connell J, et al. Research methodologies in informed consent studies involving surgical and invasive procedures: Time to re-examine? *Patient Education and Counseling* 2013;93(3):559-66. doi: DOI 10.1016/j.pec.2013.08.018
32. Craig P, Dieppe P, Macintyre S, et al. Developing and evaluating complex interventions: the new Medical Research Council guidance. *BMJ* 2008;337:a1655.

For peer review only - http://bmjopen.bmj.com/site/about/guidelines.xhtml

Table 1 Participant characteristics

	Clinical centers	
	**Responders (n=63)**	**Non-responders (n=18)**
Region (%)		
Northern England	14 (22)	5 (28)
Midland	8 (13)	0
South East England	22 (35)	10 (55)
South West England	9 (14)	0
Wales	10 (16)	3 (17)
Mean number of major colorectal resections (range)	117 (38 to 275)	90 (29 to 210)
	**Patients**	
	**Responders (n=97)**	**Non-responders (n=170)**
Mean age (range)	64 (29 to 87)	68 (29 to 88)
Female (%)	41 (42)	95 (56)
Cancer site (%)		
Rectum/anus	33 (35)	55 (32)
Left colon	34 (36)	46 (27)
Right colon	30 (29)	60 (36)
Unknown		9 (5)
IMD quintile (%)*		
1	5 (5)	27 (16)
2	13 (13)	38 (23)
3	20 (21)	24 (14)
4	20 (21)	41 (24)
5	39 (40)	23 (23)
Educational level (%)		
Basic	30 (32)	
Higher	34 (35)	
Undergraduate	16 (16)	
Postgraduate	6 (6)	
Not disclosed	11 (11)	
Marital status (%)		
Single/divorced	17 (18)	
Married/cohabiting	73 (75)	
Widowed	7 (7)	
Employment status (%)		
Employed	16 (17)	
Retired	58 (60)	
Seeking work	1 (1)	
Not working voluntarily	5 (5)	
Sickness leave	5 (5)	
Other	12 (12)	
Length of hospital stay (%)		
<2 weeks	80 (83)	
2-3 weeks	10 (10)	
3-4 weeks	3 (3)	
> 4 weeks	4 (4)	

For peer review only

*IMD: Index of Multiple Deprivation as defined by the UK Office of National Statistics at lower layer Super Output Area level for the individual. Lower quintile equates to higher deprivation

For peer review only

For peer review only - http://bmjopen.bmj.com/site/about/guidelines.xhtml

Table 2 Voting on information domains to be included in the CIS in the surgeon consensus meetings

Information domain	Voted in (N=61) n (%)	Consensus
Anastomotic leak	40 (65)	In
Conversion to open operation	38 (63)	In
Post-operative mortality	41 (62)	In
DVT/PE	36 (59)	In
Long-term survival	33 (54)	In
Stoma formation	32 (53)	In
Surgical site infection	30 (49)	Out
Cancer recurrence	29 (48)	Out
Re-operation	26 (43)	Out
Haemorrhage	25 (41)	Out
Sexual functioning	25 (41)	Out
Global quality of life	24 (40)	Out
Bowel obstruction	22 (37)	Out
Faecal incontinence	20 (34)	Out
Expected in-hospital experience	20 (34)	Out
Visceral injury	18 (29)	Out
Faecal urgency	18 (29)	Out
Stoma complications	15 (25)	Out
Physical functioning	12 (19)	Out
Re-admission	10 (17)	Out
Abandoning the operation	10 (17)	Out
Resection margins	2 (4)	Out
Lymph node yield	2 (4)	Out

For peer review only

For peer review only - http://bmjopen.bmj.com/site/about/guidelines.xhtml

Table 3 Final core information set

Information about experiences in hospital

- Expected in hospital experience including length of stay
- Peri-operative survival
- Surgical site infection
- Venous thrombo-embolism
- Anastomotic leak
- Stomata and complications
- Conversion to open operation (where appropriate)

Information about experiences after discharge

- Cancer recurrence
- Resection margins
- Long-term survival
- Quality of life including physical and sexual function, fecal incontinence and urgency

For peer review only

For peer review only - http://bmjopen.bmj.com/site/about/guidelines.xhtml

Developing core outcome and information sets for patients undergoing colorectal cancer surgery

ROUND ONE QUESTIONNAIRE FOR CLINICIANS

The following sections list some problems that can occur following CRC surgery. Please rate how essential you feel that these are:

(1) routinely reported in clinical trials of CRC surgery

(2) routinely disclosed to patients during informed consent for CRC surgery.

The descriptions are in lay terms to allow comparisons to be made with patient responses and the words in brackets represent the medical terminology. Please circle the number that best represents your opinion.

		Not essential				(please circle)				Absolutely essential
For example:										
	Clinical trials	1	2	3	4	5	6	7	8	9
	Informed consent	1	2	3	4	5	6	7	8	9

SECTION B1 SPECIFIC PROBLEMS AFTER CRC SURGERY

		Not essential				(please circle)				Absolutely essential
1	The need for a stoma – an artificial opening on the wall of the stomach/abdomen to collect food waste									
	Clinical trials	1	2	3	4	5	6	7	8	9
	Informed consent	1	2	3	4	5	6	7	8	9
2	Problems with the stoma, such as the opening becoming narrowed, dead or retracted *(stenosis, necrosis, retraction)*									
	Clinical trials	1	2	3	4	5	6	7	8	9
	Informed consent	1	2	3	4	5	6	7	8	9
3	Leakage from the join between two lengths of bowel, which may cause peritonitis, a second operation or death *(anastomotic leak)*									
	Clinical trials	1	2	3	4	5	6	7	8	9
	Informed consent	1	2	3	4	5	6	7	8	9
4	Infection inside the stomach/abdomen that may require further surgery *(deep surgical site infection)*									
	Clinical trials	1	2	3	4	5	6	7	8	9
	Informed consent	1	2	3	4	5	6	7	8	9

Figure 1. Questionnaire front sheet

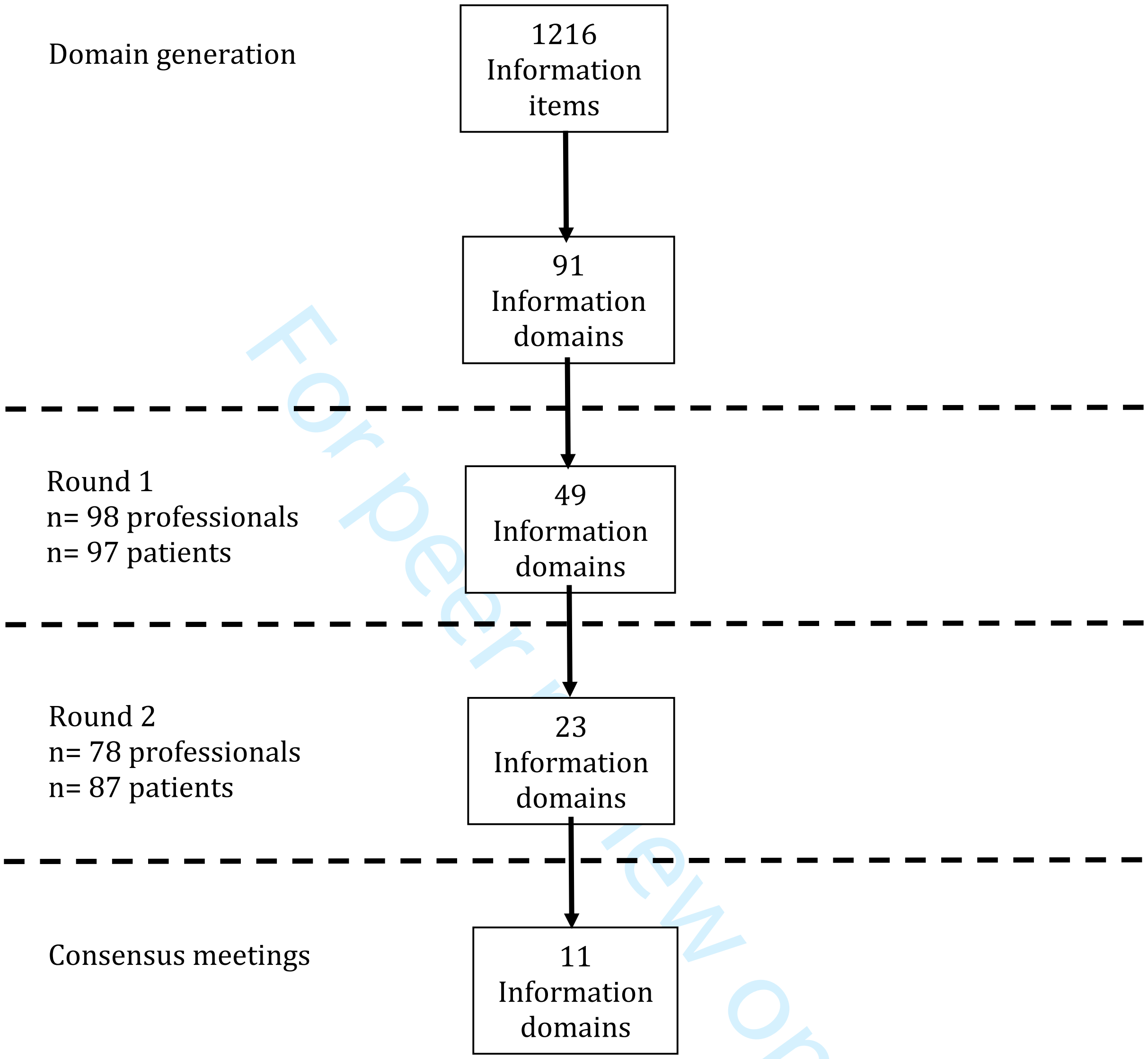
Domain generation
1216 Information items
91 Information domains
Round 1
n= 98 professionals
n= 97 patients
49 Information domains
Round 2
n= 78 professionals
n= 87 patients
23 Information domains
Consensus meetings
11 Information domains

Supplementary Table 1 Patient and professional scoring of information domains in Round 1 and 2

	Round 1				Round2			
**Information domain**	**n (%) patients rating domain low importance[a]**	**n (%) patients rating domain high importance[b]**	**n (%) HCP rating domain low importance[a]**	**n (%) HCP rating domain high importance[b]**	**n (%) patients rating domain low importance[a]**	**n (%) patients rating domain high importance[b]**	**n (%) HCP rating domain low importance[a]**	**n (%) HCP rating domain high importance[b]**
Resection margins	2(2)	88(91)	13(13)	69(70)	1(1)	82(94)	5(5)	74(75)
The need for a stoma	3(3)	84(87)	0(0)	97(98)	1(1)	82(94)	0(0)	94(96)
Distant recurrence	8(8)	81(83)	20(20)	48(48)	2(2)	74(85)	7(7)	52(53)
Local recurrence	6(6)	80(82)	21(21)	50(52)	2(2)	73(84)	8(8)	47(48)
Recurrence	7(7)	80(82)	18(18)	52(54)	1(1)	72(83)	8(8)	50(51)
Non-progression	10(10)	80(82)	18(18)	49(50)	2(2)	69(79)	7(7)	50(51)
Disease-free interval	10(10)	79(81)	30(31)	26(27)	4(5)	67(77)	15(15)	21(21)
Sphincter preservation*	14(14)	74(76)						
Lymph node harvest	14(14)	72(74)	56(58)	22(22)	3(3)	55(63)	37(38)	16(16)
Survival	12(12)	71(73)	34(35)	30(31)	6(7)	61(70)	11(11)	30(30)
Physical function	6(6)	70(72)	19(19)	39(39)	2(2)	62(71)	5(5)	25(25)
Problems with the stoma, for example leaks,unpleasant odours/noises or embarrassment	6(6)	66(68)	22(22)	51(53)	2(2)	62(71)	7(7)	37(38)
Overall quality of life	7(7)	64(66)	19(19)	46(46)	4(5)	62(71)	11(11)	42(43)
Role function	6(6)	64(66)	15(15)	49(50)	3(3)	59(68)	7(7)	33(34)
Anastomotic leak	16(16)	63(65)	0(0)	97(98)	5(6)	67(77)	0(0)	94(96)
Ability to cope emotionally with being diagnosed and treated for bowel cancer	9(9)	63(65)	28(29)	28(29)	8(9)	58(67)	18(19)	23(24)
Outlook on life after being diagnosed and treated for bowel cancer	11(11)	63(65)	35(36)	28(29)	7(8)	54(62)	20(20)	28(29)
Self-care	4(4)	62(64)	13(13)	50(52)	0(0)	59(68)	8(8)	36(37)

For peer review only

Rectal bleeding	9(9)	62(64)	32(33)	29(30)	3(3)	57(66)	17(17)	22(23)
General pain	11(11)	61(63)	16(16)	43(43)	7(8)	54(62)	11(11)	32(33)
Deep surgical site infection	18(19)	60(62)	4(5)	81(83)	6(7)	61(70)	0(0)	91(93)
Faecal incontinence	7(7)	59(61)	8(9)	65(66)	0(0)	60(69)	5(5)	67(69)
Overall health	3(3)	59(61)	27(28)	42(42)	5(6)	53(61)	15(15)	25(25)
Problems with stomata (e.g. stenosis, necrosis, retraction)	20(21)	57(59)	9(10)	58(60)	10(11)	57(66)	5(5)	62(64)
Bowel obstruction	9(9)	56(58)	19(19)	41(41)	2(2)	62(71)	6(6)	41(42)
Length of time after surgery until the bowels open	9(9)	56(58)	45(45)	16(16)	4(5)	50(57)	32(33)	12(12)
The need for extra aids to control symptoms, for example pain killers or incontinence pads	14(14)	56(58)	25(26)	35(36)	4(5)	47(54)	11(11)	22(23)
Superficial surgical site infection	17(18)	54(56)	1(2)	77(79)	6(7)	52(60)	1(1)	82(84)
Length of bowel removed	15(15)	54(56)	65(66)	8(9)				
Conversion to open operation	14(14)	53(55)	1(2)	83(85)	8(9)	48(55)	0(0)	76(78)
Diarrhoea	6(6)	52(54)	14(14)	54(56)	5(6)	46(53)	8(8)	40(41)
Faecal urgency	8(8)	51(53)	10(11)	66(67)	3(3)	52(60)	2(2)	69(70)
Constipation	8(8)	51(53)	34(35)	29(30)	6(7)	39(45)	20(20)	12(12)
Ascites	19(20)	51(53)	63(64)	9(10)				
Faecal discrimination	9(9)	50(52)	24(24)	37(37)	4(5)	41(47)	12(12)	37(38)
Length of time after surgery to start eating and drinking	17(18)	50(52)	34(35)	37(37)	7(8)	36(41)	15(15)	21(21)
Cognition	14(14)	50(52)	47(47)	10(11)	10(11)	34(39)	26(26)	11(11)
Wound dehiscence	21(22)	50(52)	24(24)	37(37)				
Enterocutaneous fistula	19(20)	50(52)	40(40)	26(27)				
Fatigue	19(20)	50(52)	41(41)	22(22)				
Length of hospital stay	14(14)	49(51)	26(27)	41(41)	7(8)	39(45)	15(15)	33(34)
Visceral injury	20(21)	47(48)	4(5)	70(71)	4(5)	48(55)	0(0)	76(78)

For peer review only - http://bmjopen.bmj.com/site/about/guidelines.xhtml

Sexual function	17(18)	47(48)	2(3)	84(86)	7(8)	48(55)	0(0)	76(78)
ileus	17(18)	47(48)	22(22)	41(41)				
Enterovisceral fistula	20(21)	47(48)	43(43)	23(23)				
Faecal frequency	8(8)	46(47)	8(9)	63(64)	2(2)	40(46)	2(2)	62(64)
Hernia	14(14)	46(47)	23(23)	37(37)				
Tenesmus	16(16)	46(47)	27(28)	38(38)				
Venous thromboembolism	15(15)	45(46)	2(3)	79(81)	4(5)	45(52)	1(1)	72(74)
Delayed wound healing	21(22)	45(46)	28(29)	31(32)				
Emotions – depression	25(26)	45(46)	33(34)	25(26)				
Renal failure	31(32)	44(45)	39(39)	18(18)				
Nausia and vomiting	11(11)	44(45)	35(36)	23(23)				
Urinary incontinence	15(15)	44(45)	40(40)	24(25)				
Operative mortality	34(35)	43(44)	5(6)	81(83)	13(15)	37(43)	2(2)	80(82)
Operative time	18(19)	43(44)	45(45)	14(14)				
Appearance/weight loss	26(27)	43(44)	51(53)	18(18)				
Shortness of breath	18(19)	43(44)	56(58)	10(11)				
Urinary frequency	14(14)	43(44)	49(50)	19(19)				
Reoperation	24(25)	42(43)	13(13)	58(60)	11(13)	33(38)	10(10)	62(64)
Incision size	26(27)	42(43)	35(36)	24(24)				
Dysphagia/regurgitation	19(20)	42(43)	63(64)	5(6)				
Bloating	12(12)	42(43)	58(60)	9(10)				
Dysuria	14(14)	42(43)	62(63)	9(10)				
Emotions – Anxiety	23(24)	42(43)	33(34)	25(26)				
Myocardial infarction	27(28)	40(41)	18(18)	50(52)	12(14)	30(34)	5(5)	45(46)
Finances	32(33)	40(41)	49(51)	22(22)				
Wound seroma	27(28)	39(40)	50(52)	13(13)				
Multi-organ failure	30(31)	39(40)	27(28)	39(39)				
Reflux	21(22)	39(40)	70(71)	5(6)				
Belching, gas or flatulence	18(19)	39(40)	71(72)	4(5)				
Arrhythmia	31(32)	38(39)	46(46)	23(23)				
Non-ischaemic/non-arrhythmic cardiac complications	29(30)	38(39)	45(45)	19(19)				

Cerebrovascular complications	25(26)	38(39)	44(44)	23(23)				
Social function	17(18)	38(39)	51(53)	13(13)				
Septicaemia	30(31)	37(38)	29(30)	30(31)				
How long it takes for a bowel movement	19(20)	37(38)	59(61)	8(9)				
postoperative haemorrhage	31(32)	36(37)	7(8)	75(77)	17(20)	32(37)	1(1)	75(76)
Pneumonia	27(28)	36(37)	9(10)	53(55)	12(14)	27(31)	3(3)	41(42)
Urinary tract infection	30(31)	35(36)	43(43)	21(21)				
Feeling faint	24(25)	35(36)	71(72)	7(8)				
Anorexia	24(25)	35(36)	52(54)	15(15)				
Insomnia	20(21)	34(35)	68(69)	9(10)				
Non-infective respiratory complications	32(33)	33(34)	39(39)	19(19)				
Thrombophlebitis	35(36)	31(32)	54(56)	13(13)				
Operative blood loss	33(34)	30(31)	31(32)	41(41)	20(23)	22(25)	13(14)	30(30)
Unplanned readmission	31(32)	30(31)	33(34)	29(30)	13(15)	16(18)	16(16)	23(24)
Allergic reactions	29(30)	29(30)	67(68)	13(13)				
Catheter complications	35(36)	29(30)	54(56)	15(15)				
Problems with periods (female)	9(9)	27(28)	59(61)	15(15)				
Rash or other skin problems	38(39)	22(23)	84(86)	1(2)				
Equipment failure	43(44)	21(22)	64(65)	11(12)				
Spiritual or faith issues	46(47)	13(13)	68(69)	8(9)				
The need for adjuvant chemotherapy#			4(5)	82(84)	1(1)	77(89)	2(2)	79(80)
Stoma reversal#			0(1)	79(81)	1(1)	73(84)	1(1)	82(84)
Expected in hosptial experience#			4(5)	80(82)	7(8)	70(80)	0(0)	80(82)
The need for bowel preparation#			11(12)	64(65)	5(6)	53(61)	7(7)	65(66)
Family risk of bowel cancer#			19(19)	42(42)				

For peer review only

[a]Low importance is defined as scoring 1-3 on a nine-point Likert scale

[b]High importance is defined as scoring 7-9 on a nine-point Likert scale

*Patient feedback resulted in the combination of domains “Sphincter preservation” into “Stoma rates” in Round 1, as these concepts were considered synonymous. “Sphincter preservation” was therefore not included in the Round 1 professional questionnaire.

#Patient feedback resulted in the addition of five information domains included in the Round 1 professional questionnaire

For peer review only

BMJ Open

The development of a core information for colorectal cancer surgery: a consensus study

Journal:	*BMJ Open*
Manuscript ID	bmjopen-2018-028623.R1
Article Type:	Original research
Date Submitted by the Author:	02-Apr-2019
Complete List of Authors:	McNair, Angus; University of Bristol, Centre for Surgical Research, Bristol Medical School: Population Health Sciences; North Bristol NHS Trust, GI Surgery<br>Whistance, Robert; University of Bristol, Centre for Surgical Research, Bristol Medical School: Population Health Sciences<br>Main, Barry; University of Bristol, Centre for Surgical Research, Bristol Medical School: Population Health Sciences<br>Forsythe, Rachael<br>Macefield, Rhiannon; University of Bristol, Centre for Surgical Research, Bristol Medical School: Population Health Sciences<br>Rees, Jonathan; University Hospitals Bristol NHS Foundation Trust, Division of Surgery,; University of Bristol, Centre for Surgical Research<br>pullyblank, anne; West of England Academic Health Science Network; North Bristol NHS Trust<br>Avery, Kerry; University of Bristol, Centre for Surgical Research, Bristol Medical School: Population Health Sciences<br>Brookes, Sara; Institute of Cancer and Genomic Sciences, University of Birmingham, Cancer Research UK Clinical Trials Unit ; University of Bristol, Population Health Sciences<br>Thomas, Michael; University Hospitals Bristol NHS Foundation Trust, Division of Surgery,<br>Sylvester, Paul; University Hospitals Bristol NHS Foundation Trust, Division of Surgery,<br>Russell, Ann; National Cancer Research Institute, Consumer Liaison Group<br>Oliver, Alfred; National Cancer Research Institute, Consumer Liaison Group<br>Morton, Dion; University of Birmingham<br>Kennedy, Robin; St Mark's Hospital, Department of Surgery<br>Jayne, David; Leeds Teaching Hospitals,<br>Huxtable, Richard; University of Bristol, Bristol Medical School: Population Health Sciences<br>Hackett, Roland; South West Cancer Network, Colorectal Site Specific Group<br>Dutton, Susan; University of Oxford, CSM<br>Coleman, Mark; University Hospitals Plymouth NHS Trust, Derriford Hospital, Department of Surgery<br>Card, Mia; University Hospitals Bristol NHS Foundation Trust, Division of Surgery,<br>Brown, Julia; University of Leeds, Consultant Leeds Institute of Clinical Trials Research

	Blazeby, Jane; University of Bristol, Centre for Surgical Research, Bristol Medical School: Population Health Sciences
**Primary Subject Heading**:	Surgery
Secondary Subject Heading:	Ethics, Oncology, Patient-centred medicine, Communication
Keywords:	Colorectal surgery < SURGERY, MEDICAL ETHICS, Gastrointestinal tumours < ONCOLOGY, Communication

SCHOLARONE™ Manuscripts

The development of a core information for colorectal cancer surgery: a consensus study

Angus GK McNair PhD FRCS[1,3], Robert N Whistance MRCS[1,2], Barry Main PhD[1,4], Rachael O Forsythe MRCS[1,2], Rhiannon Macefield[1], Jonathan Rees PhD[1], Anne M Pullyblank FRCS[3], Kerry NL Avery PhD[1], Sara T Brookes PhD[1], Michael G Thomas FRCS[5], Paul A Sylvester FRCS[5], Ann Russell[6], Alfred Oliver[6], Dion Morton FRCS[7], Robin Kennedy FRCS[8], David G Jayne FRCS[9], Richard Huxtable PhD[10], Roland Hackett[11], Susan J Dutton PhD[12], Mark G Coleman FRCS[13], Mia Card[5], Julia Brown PhD[14], Jane M Blazeby FRCS[1,2]

[1]Centre for Surgical Research, Bristol Medical School: Population Health Sciences, University of Bristol, 39 Whatley Road, Bristol, UK

[2]Division of Surgery Head and Neck, University Hospitals Bristol NHS Foundation Trust, Bristol, UK

[3]Department of General Surgery, North Bristol NHS Trust, Bristol, UK

[4]Severn School of Surgery, Bristol, UK

[5]Colorectal Surgery Unit, University Hospitals Bristol NHS Foundation Trust, Bristol, UK

[6]Colorectal Consumer Liaison Group, National Cancer Research Institute, London, UK

[7]Academic Department of Surgery, University of Birmingham, Birmingham, UK

[8]Department of Surgery, St Mark's Hospital and Academic Institute, Harrow, UK

[9]Academic Surgical Unit, St James' University Hospital NHS Trust, Leeds, UK

[10]Centre for Ethics in Medicine, University of Bristol, Bristol, UK

[11] Colorectal Site Specific Group, Somerset, Wiltshire, Avon & Gloucestershire, South West Cancer Network UK

[12]Centre for Statistics in Medicine and Oxford Clinical Trials Research Unit, Nuffield Department of Orthopaedics, Rheumatology and Musculoskeletal Sciences, University of Oxford, Oxford, UK

[13]Department of Colorectal Surgery, Plymouth Hospitals NHS Trust, Plymouth, UK

[14]Clinical Trials Research Unit, University of Leeds, Leeds, UK

Corresponding author:
Angus McNair
Centre for Surgical Research
Bristol Medical School: Population Health Sciences
University of Bristol
Canynge Hall
39 Whatley Road
Bristol
BS8 2PS United Kingdom
Email: angus.mcnair@bristol.ac.uk
Tel: +44 177 928 3495 Fax: +44 117 925 2736

Funding:
This work was supported by the MRC ConDuCT-II Hub (Collaboration and innovation for Difficult and Complex randomised controlled Trials In Invasive procedures - MR/K025643/1) and the NIHR Biomedical Research Centre at the University Hospitals Bristol NHS Foundation Trust and the University of Bristol. RNW was supported by an NIHR doctoral research fellowship. AM is funded by a Clinician Scientist Fellowship (NIHR-CS-2017-17-010) from the National Institute for Health Research. The views expressed in this publication are those of the author(s) and not necessarily those of the NHS, the National Institute for Health Research, Health Education England or the Department of Health

For peer review only - http://bmjopen.bmj.com/site/about/guidelines.xhtml

ABSTRACT

Objective: 'Core information sets' (CIS) represent baseline information, agreed by patients and professionals, to stimulate individualized patient-centred discussions. This study developed a CIS for use before colorectal cancer (CRC) surgery.

Design: Three phase consensus study: 1) Systematic literature reviews and patient interviews to identify potential information of importance to patients; 2) UK national Delphi survey of patients and professionals to rate the importance of the information; 3) International consensus meeting to agree the final CIS.

Setting: National United Kingdom CRC centres.

Participants: Purposive sampling was conducted to ensure CRC center representation based upon geographical region and caseload volume. Responses were received from 63/81(78%) centers (90 professionals). Adult patients who had undergone CRC surgery were eligible, and purposive sampling was conducted to ensure representation based on age, sex and cancer location (rectum, left and right colon). Responses were received from 97/267(35%) patients with a wide age range(29-87), equal sex ratio and cancer location. Attendees of the international Tripartite Colorectal Conference were eligible for the consensus meeting

Outcomes: Phase 1: Information of potential importance to patients was extracted verbatim and operationalized into a Delphi questionnaire. Phase 2: Patients and professionals rated the importance information on a 9-point Likert scale, and re-surveyed following group feedback. Information rated of low importance were discarded using pre-defined criteria. Phase 3: A modified nominal group technique was used to gain final consensus in separate consensus meetings with patients and professionals.

Results: Data sources identified 1216 pieces of information that informed a 100-item questionnaire. Analysis led to 46 and 23 information domains being retained after the first and second surveys, respectively. The final CIS included 11 concepts including specific surgical complications, short and long-term survival, disease recurrence, stoma and quality of life issues.

For peer review only - http://bmjopen.bmj.com/site/about/guidelines.xhtml

Conclusions: This study has established a CIS for professionals to discuss with patients before CRC surgery.

Strengths and limitations of this study

- Robust consensus methodology and transparent reporting was used to allow public scrutiny of all information included or excluded from the CIS
- Patient and professional views were given equal weighting in the consensus process
- The patient response rate was low, however, purposive sampling ensured representation of key demographics.

Keywords: Colorectal cancer, Communication, informed consent, shared decision-making

Word Count: 3236

For peer review only

For peer review only - http://bmjopen.bmj.com/site/about/guidelines.xhtml

Introduction

High-quality, patient-centered communication is a cornerstone of clinical practice in the United Kingdom and worldwide[1-4]. Such communication can facilitate shared decision making, where doctors and patients agree on a treatment plan that best fits the patient's needs, and ensure that patients' fundamental ethical right to self-determination is maintained through the process of informed consent[5]. This is particularly important in surgical oncology. For many patients with cancer, surgery offers the best chance of long-term survival but this must be balanced against short term mortality and potentially irreversible deterioration in quality of life. Understanding and weighing-up the nature and consequences of treatment, based upon personal values, is therefore an important part of the cancer journey.

There are, however, significant challenges in addressing the information needs of individual patients. The amount of information that could be discussed is vast, and it is unclear what information is critical to inform understanding in an individual. Generally patients prefer more rather than less information[6], but there is a danger of overwhelming patients with information that is not important to them, and this may reduce understanding and increase anxiety[7]. "Important" information is itself subjective, and what is important to one person may not be to another. This includes information that may or may not be relevant to the nature and consequences of the proposed surgery. Patient-led communication, where discussions are guided by the individual, is helpful but patients may lack sufficient baseline knowledge to ask important questions.

Beyond these professional and ethical responsibilities, there are additional requirements to meet legal standards of information provision for informed consent. There has been a gradual shift away from a physician-centered, paternalistic model of consent towards a "reasonable-patient" standard[5]. This aligns the process of informed consent to the patients' perspective, and requires surgeons to discuss all relevant information about a proposed treatment that an objective patient would find

For peer review only - http://bmjopen.bmj.com/site/about/guidelines.xhtml

material to making an informed decision. This model has been endorsed through the recent *Montgomery* ruling in the UK, and is common in other jurisdictions[8], but it is at once confusing and helpful in addressing patients' information needs[9]. On the one hand, it is a somewhat abstract concept that does little to help physicians tailor information to the individual[10]. Without a clear understanding of who a reasonable patient might be, the temptation exists to over disclose. On the other hand, the reasonable-patient standard might serve best if viewed as a baseline from which more meaningful, person-centered conversations develop.

One method for balancing over and under disclosure of information is to develop a "core information set" (CIS) for a specific treatment. Core information represents baseline information, determined by patients and clinicians, necessary to stimulate further patient-centered communication[11-14]. A CIS is intended for use once a treatment recommendation or decision has been made and will provide relevant information about a single intervention. This differs from the role and purpose of a decision aid, which deliberately provides information about alternative treatment choices[15]. It has the advantages of being feasible and transferable to a wide number of settings, possible to define using established health services research methodology, aligned with contemporary ethical theory, and potentially meeting the "reasonable patient" legal standard of informed consent (IC). CISs are available for oesophageal and oropharyngeal cancers [12 16].

The aim of this study is to define a CIS for CRC surgery. CRC is the third most common cancer in men and second most common in women worldwide, with an estimated incidence over 740,000 and 610,000 respectively[17]. The majority of these patients will undergo a surgical resection and methods are therefore needed to ensure that informed consent is optimized.

Methods

Study Design

For peer review only - http://bmjopen.bmj.com/site/about/guidelines.xhtml

For peer review only

This study was conducted in three phases using methods modified from the development of core outcome sets for randomized controlled trials[18]: 1) A long-list of potential information of importance before CRC surgery was identified, and categorized into domains; 2) Domains were operationalized into a questionnaire that was used to survey stakeholders' views on the importance of each domain using Delphi methods; 3) Consensus meetings with patients and professionals were used to finalize the CIS. Ethics regulatory approval was granted (UK NRES 10/H0102/82).

This study was conducted in parallel with the development of a set of outcomes to measure in CRC randomized trials, the results of which are published elsewhere[19]. The same study population was used to concurrently address this separate research question. It was hypothesized that there may be observed differences between the core outcome and information sets as they are conceptually different.

Phase 1 - Domain generation

Information about CRC surgery was identified from systematic reviews of clinical and patient reported outcome literature (published elsewhere[20 21]), supplemented by a review of patient information leaflets and interviews with patients to identify additional information not present in the published literature.

An information long-list was created, and similar information was categorized into domains by two members of the study team and a patient representative. The final domains were operationalized into questionnaire items using lay language, and piloted by patients for face validity, understanding and acceptability.

Phase 2 - Delphi consensus process

The questionnaire was sent to key stakeholders (CRC surgeons, specialist nurses and patients who had undergone surgery for CRC). Patients were essential stakeholders as they are the recipient of

For peer review only - http://bmjopen.bmj.com/site/about/guidelines.xhtml

treatment, and surgeons and nurses have an in-depth understanding of the potential impact of surgery. Family, carers and friends were not included because, although these people form part of clinical discussions, informed consent is the prerogative of an autonomous individual. Participants were welcome to consult with others when considering the importance of information domains.

Professionals were identified from UK National Health Service hospital trusts that participated in the UK National Bowel Cancer Audit. Non-probabilistic purposive sampling was conducted to ensure center variation based upon geographical region (Northern England, the Midlands, South West and South East England, and Wales), and caseload volume. Patients were recruited from University Hospitals Bristol NHS Foundation Trust, North Bristol NHS Trust and Plymouth Hospitals NHS trust. Participants were approached by post from participating centres and sent a participant information leaflet, a consent form and the questionnaire with a stamp addressed return envelope. Non-probabilistic purposive sampling was conducted to ensure representation based on age, sex and cancer site (rectum, left colon, right colon). Demographic data, including area of deprivation, marital status, employment status, educational level, were collected. Deprivation was as defined by the UK Office of National Statistics Index of Multiple Deprivation at lower layer Super Output Area level for the individual[22]. This is a combined measure of income, employment, health and disability, education, barriers to public services, crime and living environment. Educational level was defined as up to basic education (to the age of 16 or completion of the UK General Certificate of Secondary Education or equivalent), further education (subsequent qualifications to the age of 18), undergraduate and postgraduate education.

Questionnaires asked participants to rate the importance of information domains on a nine-point Likert scale, ranging from 1 (‘not essential’) to 9 (‘absolutely essential’). Information domains considered not essential after round 1 were discarded (see analyses, below). In round 2, participants were provided with feedback from round 1 in the form of their previous score for each domain and a mean score from their stakeholder group. Participants then rescored each information domain on

For peer review only - http://bmjopen.bmj.com/site/about/guidelines.xhtml

the nine-point Likert scale, and the results used to determine which domains should be retained and presented in the consensus meetings.

Professionals were also asked to rate the importance of most domains to be included as trial outcome measures in a core outcome set, the results of which are published elsewhere[19]. An example page from the questionnaire is presented in Figure 1 to demonstrate how professionals were asked both questions simultaneously. Some information did not have a corresponding trial outcome measure (e.g. “expected in hospital experience” or “family risk of bowel cancer”) and, in those instances, professionals were only asked to rate them as information. Patients were not asked separate questions about trial outcome measures because, based on feedback from the patient steering group, the two concepts were considered synonymous.

Phase 3 – Face to face consensus meetings

Separate consensus meetings were held with professionals and patients. The professional consensus meeting was conducted at the Tripartite Colorectal Meeting (meeting of the Association of Coloproctology of Great Britain and Ireland, the American Society of Colon and Rectal Surgeons, the Royal Society of Medicine, Royal Australasian College of Surgeons, Colorectal Surgical Society of Australia and New Zealand and the European Society of Coloproctology) in Birmingham, UK in 2014. The meeting was open to all members of international societies. The patient meeting was held in Bristol, UK in 2013. Attendees at this meeting were all from the UK and had completed the questionnaire surveys and responded to an invitation to attend a consensus meeting.

The retained information domains from the second survey were presented and discussed at the meetings. Anonymized voting took place to ask participants to vote each domain as either ‘In’, ‘Out’ or ‘Unsure’ using electronic keypads. Histograms and descriptive statistics were created for each domain during the meeting and displayed to the participants. Where consensus was not reached further discussion ensured and additional voting.

For peer review only - http://bmjopen.bmj.com/site/about/guidelines.xhtml

Sample size

There are no agreed methods to set the sample size for Delphi surveys or consensus meetings. Therefore, an opportunistic approach was used with the aim of obtaining approximately 100 respondents for each stakeholder group for the survey and a smaller group in which discussion could take place in the consensus meetings.

Data analyses

Information domains rated between 7-9 by over 50%, and between 1-3 by less than 15%, of respondents in round 1 were retained for round 2. Information domains not meeting these criteria were discarded. Mean scores were calculated for each retained domain to form the feedback for Round 2. Round 2 domains were retained using stricter criteria (between 7-9 by over 70%, and between 1-3 by less than 15%, of respondents). There are no agreed methods for selecting cut-off criteria within Delphi studies[23], and therefore the criteria were selected after discussion with collaborators. Domains retained after Round 2 were considered in the consensus meetings. Inclusion in the final CIS was determined at the professional consensus meeting by majority vote. In the patient consensus meeting, two rounds of voting were conducted to ensure patients adequately understood the domains. Domains that were voted for by 60% of patients were included in the CIS after initial voting. Those voted for by 40-60% were brought forward for further discussion and those voted less than 40% were discarded. In the second round, domains that achieved a two-thirds majority were included. All information domains retained from either meetings were included in the final CIS. Patients considered important information for informed consent and trial outcomes to be synonymous. Results of the patient consensus meeting are therefore presented as part of the core outcome set and are not repeated here[19].

Patient and public involvement

For peer review only - http://bmjopen.bmj.com/site/about/guidelines.xhtml

Patients collaborators were involved in developing the study design, categorizing information from data sources into domains, designing the Delphi questionnaire using patient centred language, and interpreting the clinical significance of results.

Results

Phase 1 - Domain generation

All data sources identified 1216 pieces of information on CRC surgery that were categorized into 100 domains. A summary of the results is presented in Figure 2.

Phase 2 - Delphi consensus process

A total of 63/81 (78%) CRC centers responded including 90 surgeons and 8 specialist nurses (Table 1). Centers represented all geographical regions of England and Wales, and caseload ranged from 38-275 operations per annum. Patient response rate was 97/267 (36%). The age range was wide, sex ratio equal and similar numbers of patients had rectal, left and right colonic tumors. Many patients lived in areas of low deprivation but there was an even distribution of basic and higher educational level. Professionals rated information about short-term technical outcomes of greatest importance in Round 1 including anastomotic leak, adequacy of resection margins and peri-operative mortality (supplementary Table 1). Although these issues were also important to patients, a priority was given to information about longer-term outcomes such as survival, distant recurrence and impact on quality of life. A total of 46 domains were retained for Round 2.

The response rate in Round 2 was 75% (78/104) for health professionals and 90% (87/97) for patients. The provision of feedback and more stringent cut-off criteria in Round 2 resulted in 23 domains being retained for consideration in the consensus meetings.

Phase 3 - consensus meetings

For peer review only - http://bmjopen.bmj.com/site/about/guidelines.xhtml

The professional and patient consensus meetings were attended by 61 and 14 participants respectively. Anonymized voting reached a consensus on six domains at the professionals meeting (Table 2). In initial anonymized voting at the patient consensus meeting, 11 domains were voted 'in', three were voted 'out' and nine were inconclusive. Subsequently, domains that were recognized as overlapping were combined[19]. 'Length of hospital stay' was broadened in scope to include all details of patients' pathway through hospital such as the location of the hospital and ward, anticipated length of hospital stay and follow-up arrangements. This information domain was re-named 'Expected in-hospital experiences'. A second round of voting reached a consensus on including three more domains into the CIS: 'anastomotic leak', 'stoma complications' and 'sexual functioning'. Patient and professional CIS were then combined (Table 3), creating a final list of 11 domains.

Discussion:

This study developed a CIS to inform consent for CRC surgery. A wide range of sources including published studies and patient interviews were used to identify the initial long-list of 100 information domains that could be communicated to patients. Established consensus methods were used to prioritize the views of patients and professionals to identify 23 domains of the greatest importance. Finally, consensus meetings with an international group of surgeons and UK patients agreed on the final CIS. It is recommended that the domains in the information set are discussed with all patients before CRC surgery as a baseline to improve patient understanding of expected events and outcomes of surgery and to catalyze questions relevant to the patient for further discussion.

Cancer patients' information needs have been studied extensively[6 24]. Whilst these papers raise important issues, none have examined in detail the information both patients and professionals consider essential to communicate in advance of surgery for CRC. A systematic review identified 239 studies investigating the information needs of patients[24]. It used a qualitative framework analysis to chart patients' documented information needs across studies. "Treatment-related information" was

For peer review only - http://bmjopen.bmj.com/site/about/guidelines.xhtml

For peer review only

the most commonly documented need (561 mentions (26%) out of a total 2122 in 239 studies), of which information about “risks and benefits of treatment” was the most frequent. “Rehabilitation information” (384 (18%) mentions), specifically “stoma care” was the next most frequently documented need. A Dutch cross sectional survey asked 101 surgeons (response rate 43%) to rate the importance of 12 selected items of information about risks of anastomotic leak and stomata[25]. Most surgeons reported to “always” provide information about the risk of anastomotic leak (99%), re-operation (93%) and stoma (93%). It is not clear, however, how this questionnaire was developed, and the scope was limited when compared to the 100 information domains included in this study. Nonetheless, these results triangulate well with the findings of the CIS work presented here.

Although there are no published CISs in CRC surgery, a similar concept has been developed for patients undergoing radiotherapy for rectal cancer[26]. A total of 37 information domains were identified through analysis of audio recorded clinical consultations and systematic literature reviews. Delphi surveys and consensus meetings with groups of patients and radiation oncologists gained consensus on 13 benefits and harms of treatment that should be discussed in consultations. Some of this information was found to have similar importance with surgeons and patients undergoing CRC surgery including information about local cancer control, survival, sexual and bowel function. Understandably, other information is unique to surgical therapy. Indeed a CIS has been developed for patients undergoing oesophagectomy[12], which shares several characteristics with the colorectal CIS, such as recommending discussions about in-hospital mortality and major complications. Differences in quality of life information reflect the nature of the surgeries, with the colorectal CIS including issues of defecation, whereas the esophageal CIS recommends discussing eating. In addition, patients and professionals agreed that discussing stomata was important before colorectal but not esophageal surgery where esophagostomy is infrequently required. It is possible that future CIS in other areas of surgical oncology will be developed and generic issues identified across the sets relevant to all patients with cancer.

For peer review only - http://bmjopen.bmj.com/site/about/guidelines.xhtml

Strengths and Limitations

Robust consensus methodology and established guidelines modified from the development of core outcome sets for randomized trials were used to develop this CIS, but there are some weaknesses. The large amount of information identified in Phase 1 required the grouping of information into domains. This introduces an element of subjectivity that was minimized through independent dual categorization, however, information may have been inappropriately grouped or separated. For example, the Dutch CIS for rectal cancer radiotherapy recommended the discussion of five topics around sexual function that this study had combined into one domain[26]. Of note, patients in this study had the opportunity to separate concepts out of domains in the consensus meetings but none chose to do so. Conversely, several domains were amalgamated where participants considered that they were unnecessarily detailed. In addition, patients were involved with the information categorization process and agreed on the domains in advance of the Delphi process. In phase 2, the scope of the Delphi process was limited to the UK before the CIS development process was opened internationally to professionals in Phase 3. This was done to exclude the least important information without the complexity of a multinational Delphi process, however different domains may have been brought forward for discussion at the consensus meetings if this were conducted. It will therefore be important to validate this set in other cultures.

There are no definitive guidelines on sample size and response rates for Delphi studies. The total number of participants in this study is comparatively high and there was a good representation of UK CRC surgical centers, but the response rates from patients was much lower. The effect of this on the validity of the Delphi is unclear because the methodology does not require a representative sample, but to gain a consensus among a wide range of individuals with disparate opinions. In that respect, this study achieved wide diversity based on a priori patient characteristics.

This study has identified an agreed minimum standard of information to be communicated before CRC surgery. Further research is now required to investigate methods to communicate this

For peer review only - http://bmjopen.bmj.com/site/about/guidelines.xhtml

information effectively in routine practice. This may include agenda setting in clinical encounters[27], using visual communication aids[28 29], or modifying hospital information leaflets to detail core information. Recent reviews of interventions to improve informed consent, however, showed that most were inadequately developed, without theory or conceptualisation[30 31]. Included studies were poorly designed, susceptible to bias, and outcome measures to assess interventions for IC were inconsistent. It will be necessary to pilot core information carefully, adhering to guidelines for the development of complex interventions[32], to ensure it is ready for robust evaluation in a cluster randomized trial. Further research is also needed to understand how CISs relate back to modern professional, ethical and legal standards worldwide.

In conclusion, this study developed an evidence-based CIS to communicate to patients before CRC surgery. It is not intended to replace individualized discussions with patients, but to act as a consistent starting point to catalyze further patient-centered discussions. Core information can be communicated by any professional in any healthcare setting, and form the basis of high-quality written information leaflets. This coordinated and reliable approach to information provision may help patients gain sufficient understanding to undergo surgery for CRC in a post *Montgomery* era.

Acknowledgements

We would like to thank Neil Smart and Katherine Baker for their help running the consensus meetings, and Claudette Blake for her administrative support throughout the whole project.

Data statement

Data from Phase 1 including full domain categorization is available open access. No additional data are available.

Author Contributions

Wrote the first draft of the manuscript: AGKM RNW RM JR JMB. Contributed to the writing

For peer review only - http://bmjopen.bmj.com/site/about/guidelines.xhtml

of the manuscript: AGKM RNWROF RM JR BM AMP MGT AR DM SJD JB KNLA STB JMB.

Agree with the manuscript's results and conclusions: AGKM RNW ROF BM RM JR AMP MGT PAS AR AO DM RK DGJ RHu RHa SJD MGC MC JB KNLA STB JMB. All authors have read, and confirm that they meet, ICMJE criteria for authorship.

Disclosure: AM is funded by a Clinician Scientist Fellowship (NIHR-CS-2017-17-010) from the UK National Institute for Health Research (NIHR) and supported by the NIHR Biomedical Research Centre at the University Hospitals Bristol NHS Foundation Trust and the University of Bristol. The views expressed in this publication are those of the author(s) and not necessarily those of the NHS, the National Institute for Health Research, Health Education England or the Department of Health

Conflict of Interest

None declared

Figure 1. Questionnaire front sheet

Figure 2. Summary of results

For peer review only

References

1. Epstein RM, Street RL. Patient-Centered Communication in Cancer Care: Promoting healing and reducing suffering. Bethesda, MD: National Cancer Institute, 2007.
2. Department of Health. The NHS constitution for England: Department of Health, 2009.
3. Australian Commission on Safety and Quality in Health Care. Patient centred care: Improving quality and safety through partnerships with patients and consumers. 2011. http://www.safetyandquality.gov.au/wp-content/uploads/2012/03/PCC_Paper_August.pdf (accessed 03/10/2016).
4. United States Government Publishing Office. The Patient Protection and Affordable Care Act. Washington DC: United States Government Office, 2010.
5. Spatz ES, Krumholz HM, Moulton BW. The New Era of Informed Consent: Getting to a Reasonable-Patient Standard Through Shared Decision Making. *JAMA* 2016;315(19):2063-4. doi: 10.1001/jama.2016.3070
6. Rutten LJ, Arora NK, Bakos AD, et al. Information needs and sources of information among cancer patients: a systematic review of research (1980-2003). *Patient Educ Couns* 2005;57(3):250-61. doi: 10.1016/j.pec.2004.06.006
7. Leydon GM, Boulton M, Moynihan C, et al. Cancer patients' information needs and information seeking behaviour: in depth interview study. *BMJ* 2000;320:909-13. doi: 10.1136/bmj.320.7239.909
8. King JS, Moulton BW. Rethinking informed consent: The case for shared medical decision-making. *Am J Law Med* 2006;32(4):429-501.
9. Main BG, McNair A, Blazeby JM. Informed Consent and the Reasonable-Patient Standard. *JAMA* 2016;316(9):992-3. doi: 10.1001/jama.2016.10555
10. Montgomery J, Montgomery E. Montgomery on informed consent: an inexpert decision? *J Med Ethics* 2016;42(2):89-94. doi: 10.1136/medethics-2015-102862 [published Online First: 2016/01/27]
11. Beauchamp TL, Childress JF. Respect for autonomy. Principles of Biomedical Ethics. Oxford: Oxford University Press 2001:57-112.
12. Blazeby JM, Macefield R, Blencowe NS, et al. Core information set for oesophageal cancer surgery. *Br J Surg* 2015;102(8):936-43. doi: 10.1002/bjs.9840
13. Main BG, Davies L, McNair A, et al. Bringing Informed Consent Back to Patients. *British Medical Journal* 2015. http://blogs.bmj.com/bmj/2014/08/05/barry-main-et-al-bringing-informed-consent-back-to-patients (accessed 07/04/2015).
14. Main BG, McNair AGK, Huxtable R, et al. Core information sets for informed consent to surgical interventions: baseline information of importance to patients and clinicians. *BMC Med Ethics* 2017;18(1):29. doi: 10.1186/s12910-017-0188-7 [published Online First: 2017/04/28]
15. Agoritsas T, Heen AF, Brandt L, et al. Decision aids that really promote shared decision making: the pace quickens. *BMJ* 2015;350:g7624. doi: 10.1136/bmj.g7624
16. Main BG, McNair AGK, Haworth S, et al. Core information set for informed consent to surgery for oral or oropharyngeal cancer: A mixed-methods study. *Clin Otolaryngol* 2018;43(2):624-31. doi: 10.1111/coa.13037 [published Online First: 2017/11/28]
17. Cancer IAfRo. GLOBOCAN 2012: Estimated Cancer Incidence, Mortality and Prevalence Worldwide in 2012. 2012. http://globocan.iarc.fr/Pages/fact_sheets_cancer.aspx (accessed 17/07/2015).

For peer review only - http://bmjopen.bmj.com/site/about/guidelines.xhtml

18. Williamson PR, Altman DG, Blazeby JM, et al. Developing core outcome sets for clinical trials: issues to consider. *Trials* 2012;13:132. doi: 10.1186/1745-6215-13-132
19. McNair A, Whistance RN, Forsythe RO, et al. Core Outcomes for Colorectal Cancer Surgery: A Consensus Study. *PLoS Med* 2016;13(6):e1002071
20. McNair AG, Whistance RN, Forsythe RO, et al. Synthesis and summary of patient-reported outcomes (PRO) to inform the development of a core outcome set in colorectal cancer surgery. *Colorectal Dis* 2015;17(11):O217-29
21. Whistance RN, Forsythe RO, McNair AG, et al. A systematic review of outcome reporting in colorectal cancer surgery. *Colorectal Dis* 2013;15(10):e548-60. doi: 10.1111/codi.12378
22. McLennan D, Barnes H, Noble M, et al. The English Indices of Deprivation 2010. 2011. https://www.gov.uk/government/uploads/system/uploads/attachment_data/file/6320/1870718.pdf (accessed 27/07/2015).
23. Black N, Murphy M, Lamping D, et al. Consensus development methods: a review of best practice in creating clinical guidelines. *Journal of health services research & policy* 1999;4(4):236-48.
24. van Mossel C, Leitz L, Scott S, et al. Information needs across the colorectal cancer care continuum: scoping the literature. *Eur J Cancer Care (Engl)* 2012;21(3):296-320. doi: 10.1111/j.1365-2354.2012.01340.x
25. Snijders HS, Kunneman M, Bonsing BA, et al. Preoperative risk information and patient involvement in surgical treatment for rectal and sigmoid cancer. *Colorectal Dis* 2014;16(2):O43-9. doi: 10.1111/codi.12481
26. Kunneman M, Pieterse AH, Stiggelbout AM, et al. Which benefits and harms of preoperative radiotherapy should be addressed? A Delphi consensus study among rectal cancer patients and radiation oncologists. *Radiother Oncol* 2015;114(2):212-7. doi: 10.1016/j.radonc.2014.11.034
27. Gobat N, Kinnersley P, Gregory JW, et al. What is agenda setting in the clinical encounter? Consensus from literature review and expert consultation. *Patient Educ Couns* 2015;98(7):822-9. doi: 10.1016/j.pec.2015.03.024
28. McNair AG, Brookes ST, Davis CR, et al. Communicating the results of randomized clinical trials: do patients understand multidimensional patient-reported outcomes? *J Clin Oncol* 2010;28(5):738-43.
29. Davis CR, McNair AG, Brigic A, et al. Optimising methods for communicating survival data to patients undergoing cancer surgery. *Eur J Cancer* 2010;46(18):3192-99.
30. Kinnersley P, Phillips K, Savage K, et al. Interventions to promote informed consent for patients undergoing surgical and other invasive healthcare procedures. *Cochrane Database of Systematic Reviews* 2013(7) doi: Artn Cd009445
Doi 10.1002/14651858.Cd009445.Pub2
31. Kim S, Jabori S, O'Connell J, et al. Research methodologies in informed consent studies involving surgical and invasive procedures: Time to re-examine? *Patient Education and Counseling* 2013;93(3):559-66. doi: DOI 10.1016/j.pec.2013.08.018
32. Craig P, Dieppe P, Macintyre S, et al. Developing and evaluating complex interventions: the new Medical Research Council guidance. *BMJ* 2008;337:a1655.

For peer review only - http://bmjopen.bmj.com/site/about/guidelines.xhtml

Table 1 Participant characteristics

	Clinical centers	
	**Responders (n=63)**	**Non-responders (n=18)**
Region (%)		
Northern England	14 (22)	5 (28)
Midland	8 (13)	0
South East England	22 (35)	10 (55)
South West England	9 (14)	0
Wales	10 (16)	3 (17)
Mean number of major colorectal resections (range)	117 (38 to 275)	90 (29 to 210)
	**Patients**	
	**Responders (n=97)**	**Non-responders (n=170)**
Mean age (range)	64 (29 to 87)	68 (29 to 88)
Female (%)	41 (42)	95 (56)
Cancer site (%)		
Rectum/anus	33 (35)	55 (32)
Left colon	34 (36)	46 (27)
Right colon	30 (29)	60 (36)
Unknown		9 (5)
IMD quintile (%)*		
1	5 (5)	27 (16)
2	13 (13)	38 (23)
3	20 (21)	24 (14)
4	20 (21)	41 (24)
5	39 (40)	23 (23)
Educational level (%)		
Basic	30 (32)	
Higher	34 (35)	
Undergraduate	16 (16)	
Postgraduate	6 (6)	
Not disclosed	11 (11)	
Marital status (%)		
Single/divorced	17 (18)	
Married/cohabiting	73 (75)	
Widowed	7 (7)	
Employment status (%)		
Employed	16 (17)	
Retired	58 (60)	
Seeking work	1 (1)	
Not working voluntarily	5 (5)	
Sickness leave	5 (5)	
Other	12 (12)	
Length of hospital stay (%)		
<2 weeks	80 (83)	
2-3 weeks	10 (10)	
3-4 weeks	3 (3)	
> 4 weeks	4 (4)	

For peer review only

*IMD: Index of Multiple Deprivation as defined by the UK Office of National Statistics at lower layer Super Output Area level for the individual. Lower quintile equates to higher deprivation

For peer review only

For peer review only - http://bmjopen.bmj.com/site/about/guidelines.xhtml

Table 2 Voting on information domains to be included in the CIS in the surgeon consensus meetings

Information domain	Voted in (N=61) n (%)	Consensus
Anastomotic leak	40 (65)	In
Conversion to open operation	38 (63)	In
Post-operative mortality	41 (62)	In
DVT/PE	36 (59)	In
Long-term survival	33 (54)	In
Stoma formation	32 (53)	In
Surgical site infection	30 (49)	Out
Cancer recurrence	29 (48)	Out
Re-operation	26 (43)	Out
Haemorrhage	25 (41)	Out
Sexual functioning	25 (41)	Out
Global quality of life	24 (40)	Out
Bowel obstruction	22 (37)	Out
Faecal incontinence	20 (34)	Out
Expected in-hospital experience	20 (34)	Out
Visceral injury	18 (29)	Out
Faecal urgency	18 (29)	Out
Stoma complications	15 (25)	Out
Physical functioning	12 (19)	Out
Re-admission	10 (17)	Out
Abandoning the operation	10 (17)	Out
Resection margins	2 (4)	Out
Lymph node yield	2 (4)	Out

For peer review only

For peer review only - http://bmjopen.bmj.com/site/about/guidelines.xhtml

Table 3 Final core information set

**Information about experiences in hospital**
Expected in hospital experience including length of stay
Peri-operative survival
Surgical site infection
Venous thrombo-embolism
Anastomotic leak
Stomata and complications
Conversion to open operation (where appropriate)
**Information about experiences after discharge**
Cancer recurrence
Resection margins
Long-term survival
Quality of life including physical and sexual function, fecal incontinence and urgency

For peer review only

For peer review only - http://bmjopen.bmj.com/site/about/guidelines.xhtml

Developing core outcome and information sets for patients undergoing colorectal cancer surgery

ROUND ONE QUESTIONNAIRE FOR CLINICIANS

The following sections list some problems that can occur following CRC surgery. Please rate how essential you feel that these are:

(1) routinely reported in clinical trials of CRC surgery

(2) routinely disclosed to patients during informed consent for CRC surgery.

The descriptions are in lay terms to allow comparisons to be made with patient responses and the words in brackets represent the medical terminology. Please circle the number that best represents your opinion.

		Not essential				(please circle)				Absolutely essential
For example:										
	Clinical trials	1	2	3	4	5	6	7	8	9
	Informed consent	1	2	3	4	5	6	7	8	9

SECTION B1 SPECIFIC PROBLEMS AFTER CRC SURGERY

		Not essential				(please circle)				Absolutely essential
1	The need for a stoma – an artificial opening on the wall of the stomach/abdomen to collect food waste									
	Clinical trials	1	2	3	4	5	6	7	8	9
	Informed consent	1	2	3	4	5	6	7	8	9
2	Problems with the stoma, such as the opening becoming narrowed, dead or retracted *(stenosis, necrosis, retraction)*									
	Clinical trials	1	2	3	4	5	6	7	8	9
	Informed consent	1	2	3	4	5	6	7	8	9
3	Leakage from the join between two lengths of bowel, which may cause peritonitis, a second operation or death *(anastomotic leak)*									
	Clinical trials	1	2	3	4	5	6	7	8	9
	Informed consent	1	2	3	4	5	6	7	8	9
4	Infection inside the stomach/abdomen that may require further surgery *(deep surgical site infection)*									
	Clinical trials	1	2	3	4	5	6	7	8	9
	Informed consent	1	2	3	4	5	6	7	8	9

Figure 1. Questionnaire front sheet

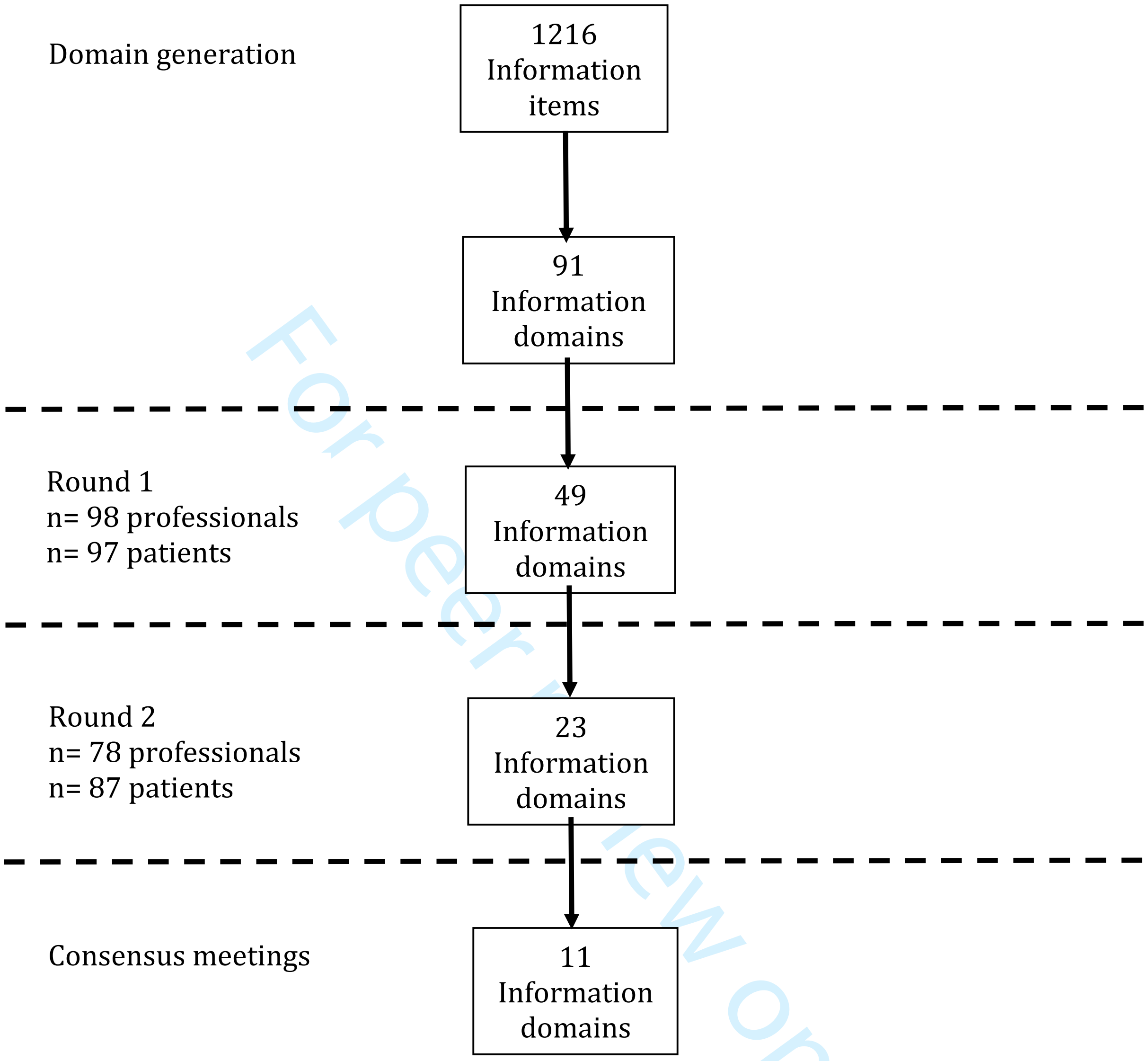

For peer review only

Supplementary Table 1 Patient and professional scoring of information domains in Round 1 and 2

	Round 1				Round2			
Information domain	n (%) patients rating domain low importance[a]	n (%) patients rating domain high importance[b]	n (%) HCP rating domain low importance[a]	n (%) HCP rating domain high importance[b]	n (%) patients rating domain low importance[a]	n (%) patients rating domain high importance[b]	n (%) HCP rating domain low importance[a]	n (%) HCP rating domain high importance[b]
Resection margins	2(2)	88(91)	13(13)	69(70)	1(1)	82(94)	5(5)	74(75)
The need for a stoma	3(3)	84(87)	0(0)	97(98)	1(1)	82(94)	0(0)	94(96)
Distant recurrence	8(8)	81(83)	20(20)	48(48)	2(2)	74(85)	7(7)	52(53)
Local recurrence	6(6)	80(82)	21(21)	50(52)	2(2)	73(84)	8(8)	47(48)
Recurrence	7(7)	80(82)	18(18)	52(54)	1(1)	72(83)	8(8)	50(51)
Non-progression	10(10)	80(82)	18(18)	49(50)	2(2)	69(79)	7(7)	50(51)
Disease-free interval	10(10)	79(81)	30(31)	26(27)	4(5)	67(77)	15(15)	21(21)
Sphincter preservation*	14(14)	74(76)						
Lymph node harvest	14(14)	72(74)	56(58)	22(22)	3(3)	55(63)	37(38)	16(16)
Survival	12(12)	71(73)	34(35)	30(31)	6(7)	61(70)	11(11)	30(30)
Physical function	6(6)	70(72)	19(19)	39(39)	2(2)	62(71)	5(5)	25(25)
Problems with the stoma, for example leaks,unpleasant odours/noises or embarrassment	6(6)	66(68)	22(22)	51(53)	2(2)	62(71)	7(7)	37(38)
Overall quality of life	7(7)	64(66)	19(19)	46(46)	4(5)	62(71)	11(11)	42(43)
Role function	6(6)	64(66)	15(15)	49(50)	3(3)	59(68)	7(7)	33(34)
Anastomotic leak	16(16)	63(65)	0(0)	97(98)	5(6)	67(77)	0(0)	94(96)
Ability to cope emotionally with being diagnosed and treated for bowel cancer	9(9)	63(65)	28(29)	28(29)	8(9)	58(67)	18(19)	23(24)
Outlook on life after being diagnosed and treated for bowel cancer	11(11)	63(65)	35(36)	28(29)	7(8)	54(62)	20(20)	28(29)
Self-care	4(4)	62(64)	13(13)	50(52)	0(0)	59(68)	8(8)	36(37)

Rectal bleeding	9(9)	62(64)	32(33)	29(30)	3(3)	57(66)	17(17)	22(23)
General pain	11(11)	61(63)	16(16)	43(43)	7(8)	54(62)	11(11)	32(33)
Deep surgical site infection	18(19)	60(62)	4(5)	81(83)	6(7)	61(70)	0(0)	91(93)
Faecal incontinence	7(7)	59(61)	8(9)	65(66)	0(0)	60(69)	5(5)	67(69)
Overall health	3(3)	59(61)	27(28)	42(42)	5(6)	53(61)	15(15)	25(25)
Problems with stomata (e.g. stenosis, necrosis, retraction)	20(21)	57(59)	9(10)	58(60)	10(11)	57(66)	5(5)	62(64)
Bowel obstruction	9(9)	56(58)	19(19)	41(41)	2(2)	62(71)	6(6)	41(42)
Length of time after surgery until the bowels open	9(9)	56(58)	45(45)	16(16)	4(5)	50(57)	32(33)	12(12)
The need for extra aids to control symptoms, for example pain killers or incontinence pads	14(14)	56(58)	25(26)	35(36)	4(5)	47(54)	11(11)	22(23)
Superficial surgical site infection	17(18)	54(56)	1(2)	77(79)	6(7)	52(60)	1(1)	82(84)
Length of bowel removed	15(15)	54(56)	65(66)	8(9)				
Conversion to open operation	14(14)	53(55)	1(2)	83(85)	8(9)	48(55)	0(0)	76(78)
Diarrhoea	6(6)	52(54)	14(14)	54(56)	5(6)	46(53)	8(8)	40(41)
Faecal urgency	8(8)	51(53)	10(11)	66(67)	3(3)	52(60)	2(2)	69(70)
Constipation	8(8)	51(53)	34(35)	29(30)	6(7)	39(45)	20(20)	12(12)
Ascites	19(20)	51(53)	63(64)	9(10)				
Faecal discrimination	9(9)	50(52)	24(24)	37(37)	4(5)	41(47)	12(12)	37(38)
Length of time after surgery to start eating and drinking	17(18)	50(52)	34(35)	37(37)	7(8)	36(41)	15(15)	21(21)
Cognition	14(14)	50(52)	47(47)	10(11)	10(11)	34(39)	26(26)	11(11)
Wound dehiscence	21(22)	50(52)	24(24)	37(37)				
Enterocutaneous fistula	19(20)	50(52)	40(40)	26(27)				
Fatigue	19(20)	50(52)	41(41)	22(22)				
Length of hospital stay	14(14)	49(51)	26(27)	41(41)	7(8)	39(45)	15(15)	33(34)
Visceral injury	20(21)	47(48)	4(5)	70(71)	4(5)	48(55)	0(0)	76(78)

For peer review only - http://bmjopen.bmj.com/site/about/guidelines.xhtml

Sexual function	17(18)	47(48)	2(3)	84(86)	7(8)	48(55)	0(0)	76(78)
ileus	17(18)	47(48)	22(22)	41(41)				
Enterovisceral fistula	20(21)	47(48)	43(43)	23(23)				
Faecal frequency	8(8)	46(47)	8(9)	63(64)	2(2)	40(46)	2(2)	62(64)
Hernia	14(14)	46(47)	23(23)	37(37)				
Tenesmus	16(16)	46(47)	27(28)	38(38)				
Venous thromboembolism	15(15)	45(46)	2(3)	79(81)	4(5)	45(52)	1(1)	72(74)
Delayed wound healing	21(22)	45(46)	28(29)	31(32)				
Emotions – depression	25(26)	45(46)	33(34)	25(26)				
Renal failure	31(32)	44(45)	39(39)	18(18)				
Nausia and vomiting	11(11)	44(45)	35(36)	23(23)				
Urinary incontinence	15(15)	44(45)	40(40)	24(25)				
Operative mortality	34(35)	43(44)	5(6)	81(83)	13(15)	37(43)	2(2)	80(82)
Operative time	18(19)	43(44)	45(45)	14(14)				
Appearance/weight loss	26(27)	43(44)	51(53)	18(18)				
Shortness of breath	18(19)	43(44)	56(58)	10(11)				
Urinary frequency	14(14)	43(44)	49(50)	19(19)				
Reoperation	24(25)	42(43)	13(13)	58(60)	11(13)	33(38)	10(10)	62(64)
Incision size	26(27)	42(43)	35(36)	24(24)				
Dysphagia/regurgitation	19(20)	42(43)	63(64)	5(6)				
Bloating	12(12)	42(43)	58(60)	9(10)				
Dysuria	14(14)	42(43)	62(63)	9(10)				
Emotions – Anxiety	23(24)	42(43)	33(34)	25(26)				
Myocardial infarction	27(28)	40(41)	18(18)	50(52)	12(14)	30(34)	5(5)	45(46)
Finances	32(33)	40(41)	49(51)	22(22)				
Wound seroma	27(28)	39(40)	50(52)	13(13)				
Multi-organ failure	30(31)	39(40)	27(28)	39(39)				
Reflux	21(22)	39(40)	70(71)	5(6)				
Belching, gas or flatulence	18(19)	39(40)	71(72)	4(5)				
Arrhythmia	31(32)	38(39)	46(46)	23(23)				
Non-ischaemic/non-arrhythmic cardiac complications	29(30)	38(39)	45(45)	19(19)				

Cerebrovascular complications	25(26)	38(39)	44(44)	23(23)				
Social function	17(18)	38(39)	51(53)	13(13)				
Septicaemia	30(31)	37(38)	29(30)	30(31)				
How long it takes for a bowel movement	19(20)	37(38)	59(61)	8(9)				
postoperative haemorrhage	31(32)	36(37)	7(8)	75(77)	17(20)	32(37)	1(1)	75(76)
Pneumonia	27(28)	36(37)	9(10)	53(55)	12(14)	27(31)	3(3)	41(42)
Urinary tract infection	30(31)	35(36)	43(43)	21(21)				
Feeling faint	24(25)	35(36)	71(72)	7(8)				
Anorexia	24(25)	35(36)	52(54)	15(15)				
Insomnia	20(21)	34(35)	68(69)	9(10)				
Non-infective respiratory complications	32(33)	33(34)	39(39)	19(19)				
Thrombophlebitis	35(36)	31(32)	54(56)	13(13)				
Operative blood loss	33(34)	30(31)	31(32)	41(41)	20(23)	22(25)	13(14)	30(30)
Unplanned readmission	31(32)	30(31)	33(34)	29(30)	13(15)	16(18)	16(16)	23(24)
Allergic reactions	29(30)	29(30)	67(68)	13(13)				
Catheter complications	35(36)	29(30)	54(56)	15(15)				
Problems with periods (female)	9(9)	27(28)	59(61)	15(15)				
Rash or other skin problems	38(39)	22(23)	84(86)	1(2)				
Equipment failure	43(44)	21(22)	64(65)	11(12)				
Spiritual or faith issues	46(47)	13(13)	68(69)	8(9)				
The need for adjuvant chemotherapy#			4(5)	82(84)	1(1)	77(89)	2(2)	79(80)
Stoma reversal#			0(1)	79(81)	1(1)	73(84)	1(1)	82(84)
Expected in hosptial experience#			4(5)	80(82)	7(8)	70(80)	0(0)	80(82)
The need for bowel preparation#			11(12)	64(65)	5(6)	53(61)	7(7)	65(66)
Family risk of bowel cancer#			19(19)	42(42)				

For peer review only - http://bmjopen.bmj.com/site/about/guidelines.xhtml

[a]Low importance is defined as scoring 1-3 on a nine-point Likert scale

[b]High importance is defined as scoring 7-9 on a nine-point Likert scale

*Patient feedback resulted in the combination of domains “Sphincter preservation” into “Stoma rates” in Round 1, as these concepts were considered synonymous. “Sphincter preservation” was therefore not included in the Round 1 professional questionnaire.

#Patient feedback resulted in the addition of five information domains included in the Round 1 professional questionnaire

For peer review only
